# Beta Adrenergic Overstimulation Impaired Vascular Contractility via Actin-Cytoskeleton Disorganization in Rabbit Cerebral Artery

**DOI:** 10.1371/journal.pone.0043884

**Published:** 2012-08-20

**Authors:** Hyoung Kyu Kim, Won Sun Park, Mohamad Warda, So Youn Park, Eun A. Ko, Min Hee Kim, Seung Hun Jeong, Hye-Jin Heo, Tae-Hoon Choi, Young-Won Hwang, Sun-Il Lee, Kyung Soo Ko, Byoung Doo Rhee, Nari Kim, Jin Han

**Affiliations:** 1 National Research Laboratory for Mitochondrial Signaling, Department of Physiology, College of Medicine, Cardiovascular and Metabolic Disease Center, Inje University, Busan, Korea; 2 Department of Physiology, Kangwon National University School of Medicine, Chuncheon, Korea; 3 Department of Biochemistry, Faculty of Veterinary Medicine, Cairo University, Cairo, Egypt; 4 Department of Pharmacology, College of Medicine and Medical Research Center for Ischemic Tissue Regeneration, Pusan National University, Busan, Korea; 5 Department of Medicine, Section of Pulmonary, Critical Care and Sleep Medicine, University of Illinois at Chicago, Chicago, Illinois, United States of America; 6 Department of Physical Education, Andong Science College, Andong, Korea; 7 Department of Neurosurgery, College of Medicine, Inje University, Busan Paik Hospital, Busan, Korea; INRCA, Italy

## Abstract

**Background and Purpose:**

Beta adrenergic overstimulation may increase the vascular damage and stroke. However, the underlying mechanisms of beta adrenergic overstimulation in cerebrovascular dysfunctions are not well known. We investigated the possible cerebrovascular dysfunction response to isoproterenol induced beta-adrenergic overstimulation (ISO) in rabbit cerebral arteries (CAs).

**Methods:**

ISO was induced in six weeks aged male New Zealand white rabbit (0.8–1.0 kg) by 7-days isoproterenol injection (300 μg/kg/day). We investigated the alteration of protein expression in ISO treated CAs using 2DE proteomics and western blot analysis. Systemic properties of 2DE proteomics result were analyzed using bioinformatics software. ROS generation and following DNA damage were assessed to evaluate deteriorative effect of ISO on CAs. Intracellular Ca^2+^ level change and vascular contractile response to vasoactive drug, angiotensin II (Ang II), were assessed to evaluate functional alteration of ISO treated CAs. Ang II-induced ROS generation was assessed to evaluated involvement of ROS generation in CA contractility.

**Results:**

Proteomic analysis revealed remarkably decreased expression of cytoskeleton organizing proteins (e.g. actin related protein 1A and 2, α-actin, capping protein Z beta, and vimentin) and anti-oxidative stress proteins (e.g. heat shock protein 9A and stress-induced-phosphoprotein 1) in ISO-CAs. As a cause of dysregulation of actin-cytoskeleton organization, we found decreased level of RhoA and ROCK1, which are major regulators of actin-cytoskeleton organization. As functional consequences of proteomic alteration, we found the decreased transient Ca^2+^ efflux and constriction response to angiotensin II and high K^+^ in ISO-CAs. ISO also increased basal ROS generation and induced oxidative damage in CA; however, it decreased the Ang II-induced ROS generation rate. These results indicate that ISO disrupted actin cytoskeleton proteome network through down-regulation of RhoA/ROCK1 proteins and increased oxidative damage, which consequently led to contractile dysfunction in CA.

## Introduction

β-adrenergic receptor (βAR) stimulation is a critical physiological mechanism for robust “fight or flight response”. However, overstimulation of βAR cause pathological left ventricular hypertrophy (LVH), which is a potent, independent predictor of cardiovascular diseases including stroke, coronary heart disease and heart failure [1], [2]. Compared with well established pathological event of βAR stimulation in heart, its effect on vasculature, especially cerebrovasculature, is still unknown.

Isoproterenol (ISO) is a synthetic catecholamine that is widely used for stimulation of all subtypes of βAR in cell [3] and animal model [4]. In the cultured cells, ISO-induced βAR stimulation activated ERK in cardiomyocytes [5] and astrocytes via PKA pathway [6]. In the rat aorta, 7 days of ISO treatment induced endothelial dysfunction and increased vasoconstriction [7]. In our previous studies, we demonstrated that ISO-βAR stimulation is associated with the modulation of Ca^2+^-activated K^+^, inward rectifier K^+^, and voltage-dependent K^+^ channels in coronary arterial smooth muscle cells, which suggested functional modification of arterial smooth muscle cells during βAR stimulation[8], [9], [10]. We also found that ISO-βAR overstimulation disrupted the signaling of Ras/Raf/ERK cascades and highly increased activation of ERK in isoproterenol treated cerebral artery(CA)[4]. Since the Ras/Raf/ERK cascade is an important regulatory mechanism for vascular contractility, our previous findings suggested that βAR overstimulation is involved in cerebrovascular events [11], [12], [13]. However, functional consequences and responsible proteomic alteration of the ISO-βAR stimulation in cerebrovasculature were not evaluated.

Therefore, we investigated the effect of βAR stimulation on cerebrovasculature using isoproterenol injected rabbit model. We tested whether βAR stimulation caused cerebrovascular damage then identified the proteomic alteration of CA and constructed protein interaction map of CA in βAR stimulation. Based on the proteomics data, we further demonstrated that βAR stimulation modified CA contractility through modulation of Ca^2+^ mobility and ROS generation.

## Materials and Methods

### Ethics Statement

All experimental procedures were approved by the Institutional Review Board of Animals, Inje University College of Medicine (approval number: 2011-062). All surgery was performed under sodium pentobarbital anesthesia, and all efforts were made to minimize suffering.

### Animals

Six weeks aged, male New Zealand white rabbits (0.8–1.0 kg) were purchased from the Orient Bio Inc. (Seongnam, Gyeonggi-do, Korea). Vehicle (0.9% saline 1 ml/kg body weight, i.v., n = 26) or isoproterenol (300 μg/kg body weight, i.v., n = 28) was infused once daily as a bolus injection [7], [10], [14], [15]. After a 7-day administration, isoproterenol-induced βAR stimulation (ISO) on model animal was evaluated by measuring the heart-to-body weight ratio and blood pressure as previously described [8], [9]. None of isoproterenol injected rabbit was dead before sacrifice.

### Cell and tissue preparation

Enzymatic isolation of CA single smooth muscle cells (SMCs) was performed as previously described [8], [9]. In detail, rabbit brains of Con and ISO model were isolated and placed in ice-cold (4°C) isolation normal tyrode (NT) solution containing 143 mM NaCl, 5.4 mM KCl, 1.8 mM CaCl_2_, 0.5 mM MgCl_2_, 5.5 mM glucose, and 5 mM HEPES (pH 7.4) adjusted with NaOH. The middle cerebral artery was dissected from the brain and disbranched. The isolated CA was then placed into Ca^2+^-free isolation solution containing 1.5 mg/ml papain, 1 mg/ml dithioerythreitol, and 1 mg/ml bovine serum albumin (BSA) for 10 min at 37°C, and then transferred into Ca^2+^-free isolation solution containing 1 mg/ml collagenase, 1 mg/ml hyaluronidase, and 1 mg/ml BSA for 8 min at 37°C. The enzyme-treated CA was washed three times in ice-cold isolation solution for 2 min. Finally, the CA was gently agitated using a polished glass pipette to obtain single SMCs.

### Pressurized arterial experiment

To assess functional modifications of ISO-CAs, we assessed drug-specific contraction in response to high K^+^ (KCl 60 mM) and angiotensin II (Ang II) in endothelium-denuded CA. Arterial diameter and drug-specific responses were assessed as previously described [9]. Briefly, the isolated middle cerebral artery (n = 4 in each group) was cannulated at both ends with micropipettes, secured with nylon monofilament suture and placed in a specially designed, custom-built chamber. The arteries were maintained in no-flow state and held at a constant intraluminal pressure of 60 mmHg. The extraluminal diameter of the artery was measured with a video edge detector (Crescent Electronics, Sandy, Utah, USA). High K^+^-induced arterial contraction was measured in 60 mM of KCl in intraluminal solution [16]. Ang II-induced arterial contraction was measured in dose dependent manner over concentration range from 1×10^−9^ to 1×10^−4^ M of Ang II in intraluminal solution. Percentage of contraction of each sample was compared to non-treated basal vessel diameter.

**Table 1:** Hemodynamic characterization of experimental animals.

	Con	ISO
**Body Weight (kg)**	1.66±0.08	1.78±0.06
**Heart Weight (g)**	**7.08±0.21**	8.72±0.64*
**Heart/Body weight (%)**	**0.43±0.01**	0.49±0.02*
**Blood pressure (mmHg)**		
Systolic	122.66±1.13	119.46±2.07
Diastolic	58.41±3.19	54.20±7.54
Mean arterial pressure	82.08±3.93	81.00±3.82
**Heart Rate (beat/min)**	212.33±18.80	256.20±7.83

Means ± SE (n = 5) *P<0.05, student T-test.

### Angiotensin II-induced intracellular Ca^2+^ release measurement

Ang II-induced intracellular Ca^2+^ release rates were measured in the isolated SMCs of Con (n = 4) and ISO-CA (n = 4) using the membrane-permeant (acetoxymethyl ester) form of the Ca^2+^-sensitive fluorescent dye Fura 2 (Fura 2-AM) and photomultipliers as previously described [17]. Enzymatically isolated SMCs (n = 4 in each group) were loaded by incubation in 3 μM Fura 2-AM for 30 min at 37°C, and the extracellular Fura 2-AM (Invitrogen, USA) was then rinsed off with normal Tyrode solution. Monochromatic excitation light (355 nm) was delivered to the cell using a filter wheel (Life Science Resources, Cambridge, UK) via a liquid light guide and an oil-immersion objective lens (X40, NA 1.3; Nikon). The light emitted through an aperture slightly larger than the cell was measured simultaneously at 340 and 380 nm, and Ca^2+^ concentration was estimated from the ratio of the fluorescence signals (340/380) obtained from the two photomultipliers (Life Science Resources) [17]. After 120 second stabilization, angiotensin II (1 μm/L)-induced intracellular Ca^2+^ level ([Ca^2+^]_i_) elevation were measured. Difference between basal (absence of Ang II)-to-peak (presence of Ang II) fluorescence ratio in each group was compared to the calculated quantitative group data. At the same time, fluorescence images of calcium transient in Fluo-4AM stained (Invitrogen, USA, 1 μM, incubation in 30 min at 37°C) Con and ISO SMCs were acquired using confocal microscope LSM 510 (Carl Zeiss, German). All experiments were carried out at room temperature (22–25°C).

**Figure 1:**
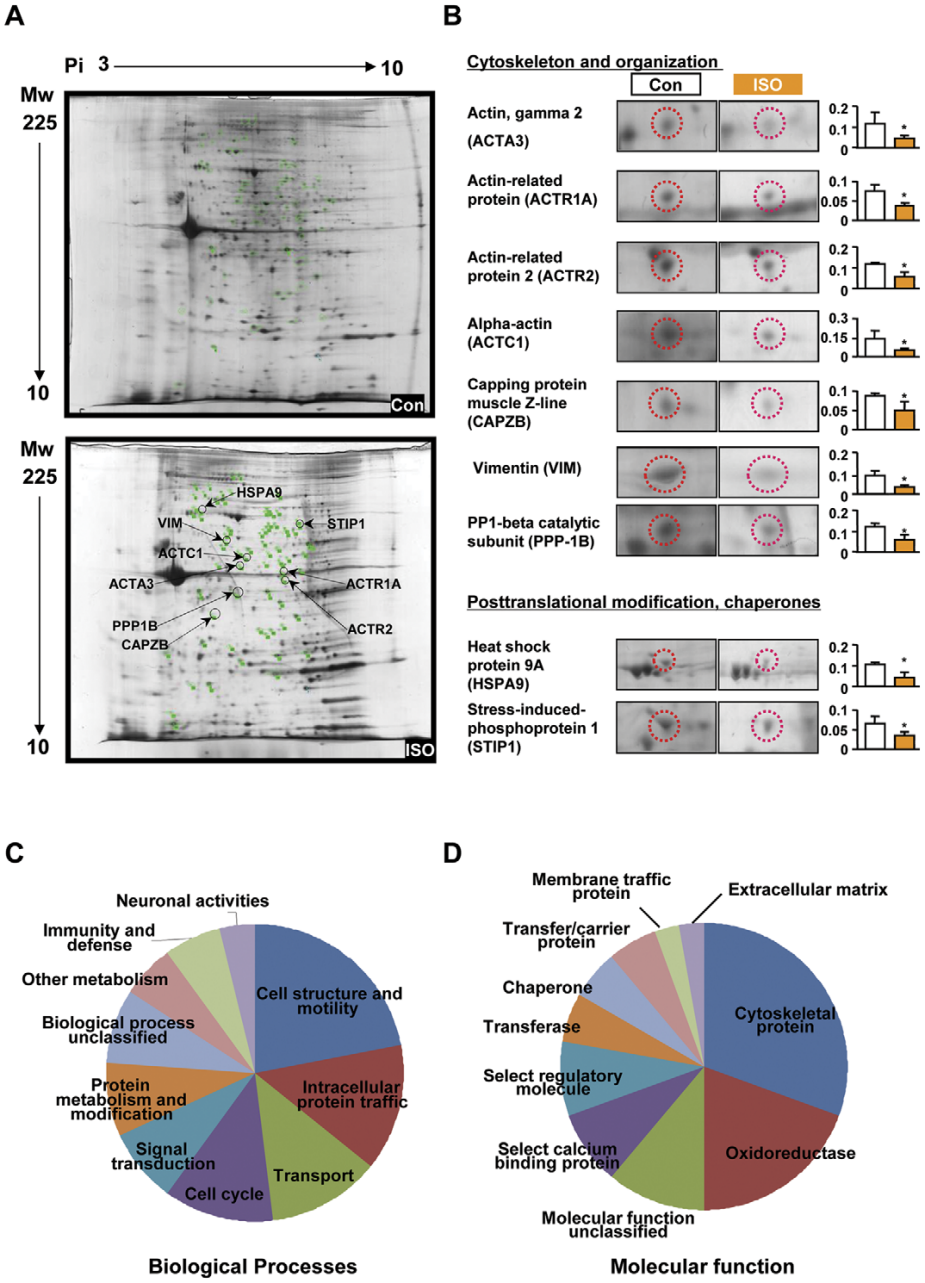
Comparative two dimensional gel electrophoresis (2DE) analysis of control and ISO-CAs. A. 2DE gel images of control (up) and ISO-CAs (down) with proteins of interest annotated. B. Enlarged spot images and histograms of significantly (n = 3 in each group, Student's t-test, *P<0.05 vs. control) altered protein expression. The protein spots were selected from ‘Cytoskeleton and organization’ and ‘Posttranslational modification, chaperones’ categories. C. Major biological processes of identified proteins. D. Major molecular function of identified proteins. Proteome categorized by Protein ANalysis THrough Evolutionary Relationships (PANTHER) algorithm.

**Table 2:** Functional category and expression changes of identified proteins.

Identification & functional category	Relative protein level in ISO	p-value	NCBI GI
**Amino acid transport and metabolism**			
Glutamate dehydrogenase (GLUD1)	0.59	0.032	51863477
**Cell cycle control, cell division, chromosome** **partitioning**			
Vimentin (VIM)	0.36	0.048	860908
Septin (SEPT8)	0.34	0.009	27448554
**Cytoskeleton**			
GRIP1 associated protein 1 (GRIPAP1)	0.29	0.045	74196422
WD repeat-containing protein 1 isoform 8 (WDR1)	0.56	0.038	114593211
Coronin-1B (CORO1B)	0.37	0.048	54035918
Actin-related protein 1 (ACTR1A)	0.5	0.043	381964
Actin-related protein 2 homolog (ACTR2)	0.47	0.013	29126784
Alpha-actin (ACTC1)	0.37	0.025	49870
Capping protein (actin filament) muscle Z-line (CAPZB)	0.55	0.05	119615295
Actin, alpha 2, smooth muscle (ACTA20	0.54	0.023	178027
**Energy production and conversion**			
NADH dehydrogenase (ubiquinone) Fe-S protein 1 (NDUFS1)	2.06	0.039	53850628
NADH dehydrogenase (ubiquinone) Fe-S protein 8 (NDUFS8)	0.39	0.034	157821497
Aldehyde dehydrogenase 1A2 isoform 1 (RALDH2)	0.37	0.048	50369993
Aldehyde dehydrogenase family 1 member A1 (ALDH1A1)	0.38	0.025	42558920
Aldehyde dehydrogenase, mitochondrial precursor (ALDH2)	0.54	0.033	73995214
Isocitrate dehydrogenase 1 (NADP+) (IDH1)	0.74	0.046	13928690
**Extracellular structures**			
Alpha-2 collagen type VI (COL6A2)	0.51	0.044	49907
**General function prediction only**			
Moesin (MSN)	0.32	0.031	13540689
**Intracellular trafficking, secretion, and vesicular transport**			
Annexin VI isoform 1 (ANX6)	0.38	0.044	71773329
Annexin A1 (Annexin I) (ANXA1)	0.59	0.033	1703316
**Nucleotide transport and metabolism**			
Dihydropyrimidinase-like 2 (DPYSL2)	0.42	0.049	115496400
**Posttranslational modification, protein turnover, chaperones**			
Heat shock protein 9A, mortalin (HSPA9)	0.42	0.032	6754256
Stress-induced-phosphoprotein 1 (STIP1)	0.55	0.047	73983760
Protein disulfide isomerase family A, member 3, (PDIA3)	0.42	0.033	119597640
Chaperonin containing TCP1, subunit 2 (beta), (CCT2)	0.49	0.029	119617634
GDP dissociation inhibitor 2 (GDI2)	0.37	0.039	40254781
Elongation factor 1-gamma (EF1G)	0.25	0.021	232037
Glutathione-S-transferase, mu 5 (GSTM5)	1.98	0.046	25282395
**Signal transduction mechanisms**			
EH-domain containing 1, isoform CRA_a (EHD1)	0.73	0.041	119594723
Serine/threonine-protein phosphatase PP1-beta catalytic subunit (PP-1B) (SPDYA)	0.49	0.037	109102505
**Translation, ribosomal structure and biogenesis**			
Albumin (ALB)	0.46	0.029	30794280

### Ang II-induced ROS generation assay

The Ang II-induced ROS production was assessed using reactive oxygen species (ROS) indicator CM-H_2_DCF-DA (Invitrogen, USA). Fluorescence excitation and emission wave lengths were ∼492–495 and 517–527 nm, respectively. Enzymatically isolated SMCs were incubated with NT solution containing 10 μM CM-H_2_DCF-DA for 30 min at 37°C. The cells were washed twice and loaded on the perfusion chamber of confocal microscope. After 5 min of stabilization, angiotensin II (1 μm/L)-induced ROS generation was measured for 20 min. ROS levels were defined as the ratio between the mean fluorescence of cells before and after the treatment with Ang II.

**Figure 2:**
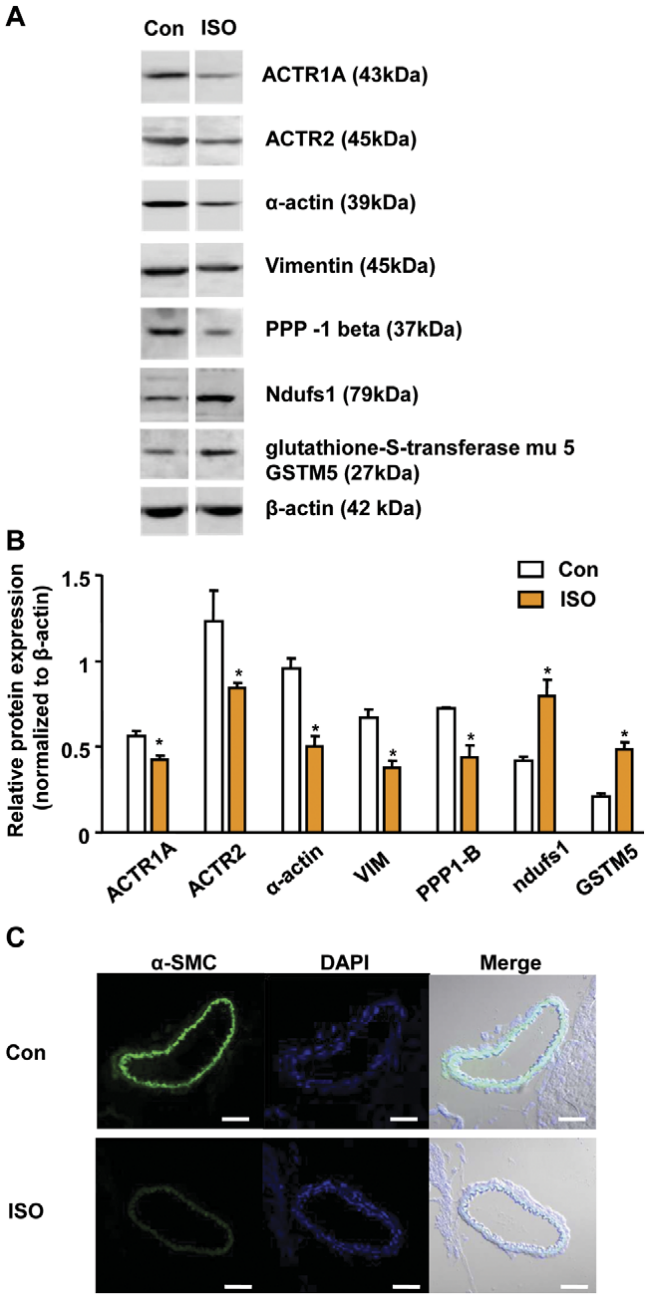
Western blot analysis of selected proteins from control and ISO-CAs. A. Representative western blot images of five down-regulated major cytoskeleton related proteins and two up-regulated non-cytoskeletal proteins. B. The relative expression rate of target proteins in control and ISO-CAs was analyzed by densitometry (n = 3 in each group, Student's t-test, *P<0.05 vs. control). C. IHC image of α SMC. C. α-smooth muscle actin expression was determined by Immunohistochemistry in Con and ISO-CAs. Sliced cerebral artery tissues were co-stained with DAPI and α-actin specific antibody. Scale bar: 50 μm.

### Superoxide production assay

We further measured the Ang II-independent ROS generation in CA-SMCs. NADPH-dependent O_2_^-^ production by CA homogenates was measured using SOD-inhibitable cytochrome c reduction assay as previously described [18]. ISO- (n = 6) and control-CA (n = 6) homogenates (final concentration 1 mg/mL) were distributed in 96-well flat-bottom plates (final volume 200 µL/well). Cytochrome c (500 µmol/L) and NADPH (100 µmol/L) were added in the presence or absence of superoxide dismutase (SOD, 200 U/mL) and incubated at room temperature for 30 minutes. Cytochrome c reduction was measured by reading absorbance at 550 nm on a microplate reader. O_2_^-^ production in nmol/mg protein was calculated from the difference between absorbance with or without SOD and the extinction coefficient, ΔE_550_ = 2.1×10^4^ M^−1^cm^−1^, for change of ferricytochrome c to ferrocytochrome c. Superoxide production was expressed in units of ‘O2- nmol/mg protein’.

**Figure 3:**
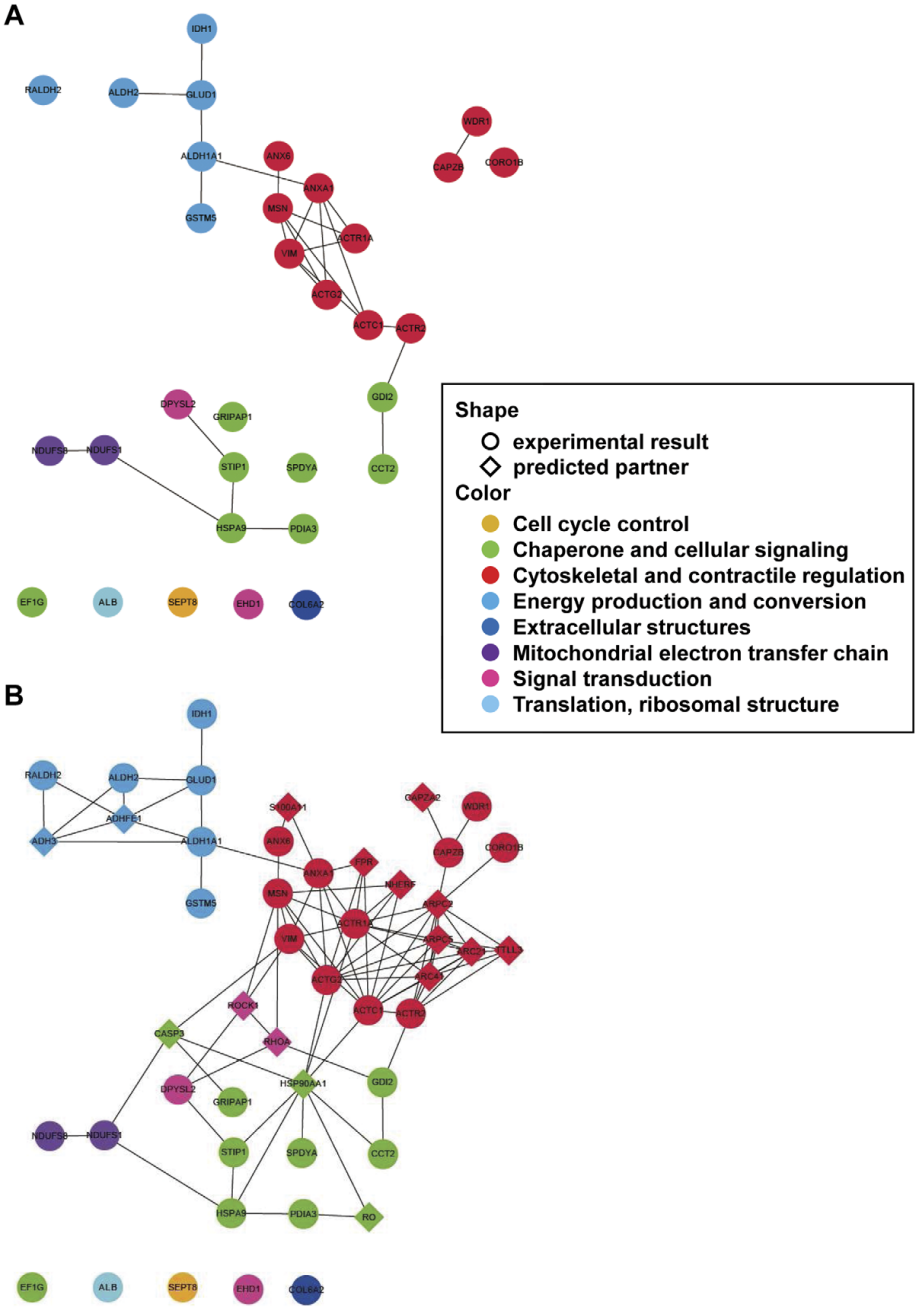
Functional association network analysis of 2D MS identified proteins. A. Protein–protein interactions among the 30 identified proteins were analyzed using STRING 8.0. Twelve of the proteins were functionally associated with the major cytoskeletal network. B. Extended network prediction provides possible interacting partners of the subjected proteins. ACTC1 =  actin, alpha cardiac muscle 1; ACTG2 =  actin, gamma-enteric smooth muscle; ACTR1A  =  alpha-centractin (centractin); ACTR2 =  actin-like protein 2 (actin-related protein 2); ADH3 =  alcohol dehydrogenase 1B; ADHFE1 =  alcohol dehydrogenase, iron containing, 1; ALDH1A1 =  retinal dehydrogenase 1; ALDH2 =  aldehyde dehydrogenase, mitochondrial precursor; ANX6  =  annexin A6; ANXA1 =  annexin A1; ARC21 =  actin-related protein 2/3 complex subunit 3; ARC41 =  actin-related protein 2/3 complex subunit 1B; ARPC2 =  actin-related protein 2/3 complex subunit 2; ARPC5 =  actin-related protein 2/3 complex subunit 5; CAPZA2 =  F-actin capping protein subunit alpha-2; CAPZB  =  F-actin capping protein subunit beta; CASP3 =  caspase-3 precursor; CCT2 =  T-complex protein 1 subunit beta; CORO1B  =  coronin-1B; DPYSL2 =  dihydropyrimidinase-related protein 2; FPR  =  fMet-Leu-Phe receptor; GLUD1 =  glutamate dehydrogenase 1, mitochondrial precursor; GRIPAP1 =  GRIP1-associated protein 1; GSTM5 =  glutathione S-transferase Mu 5; HSP90AA1 =  heat shock protein HSP 90-alpha; HSPA9 =  stress-70 protein, mitochondrial precursor; MSN  =  moesin (membrane-organizing extension spike protein); NDUFS1 =  NADH-ubiquinone oxidoreductase 75 kDa subunit; NDUFS8 =  NADH dehydrogenase iron-sulfur protein 8; NDUFV2 =  NADH dehydrogenase flavoprotein 2; NHERF  =  ezrin-radixin-moesin-binding phosphoprotein 50; PDIA3 =  protein disulfide-isomerase A3 precursor; RALDH2 =  retinal dehydrogenase 2; RO  =  calreticulin precursor (CRP55) (calregulin); RhoA  =  Ras homolog gene family, member A; ROCK1 =  Rho-associated, coiled-coil containing protein kinase 1; S100A11 =  protein S100-A11 (S100 calcium-binding protein A11) (calgizzarin) (MLN 70); SPDYA  =  serine/threonine-protein phosphatase PP1-beta catalytic subunit; STIP1 =  stress-induced-phosphoprotein 1; TTLL3 =  tubulin–tyrosine ligase-like protein 3; VIM  =  vimentin; WDR1 =  WD repeat protein 1 (actin-interacting protein 1).

**Figure 4:**
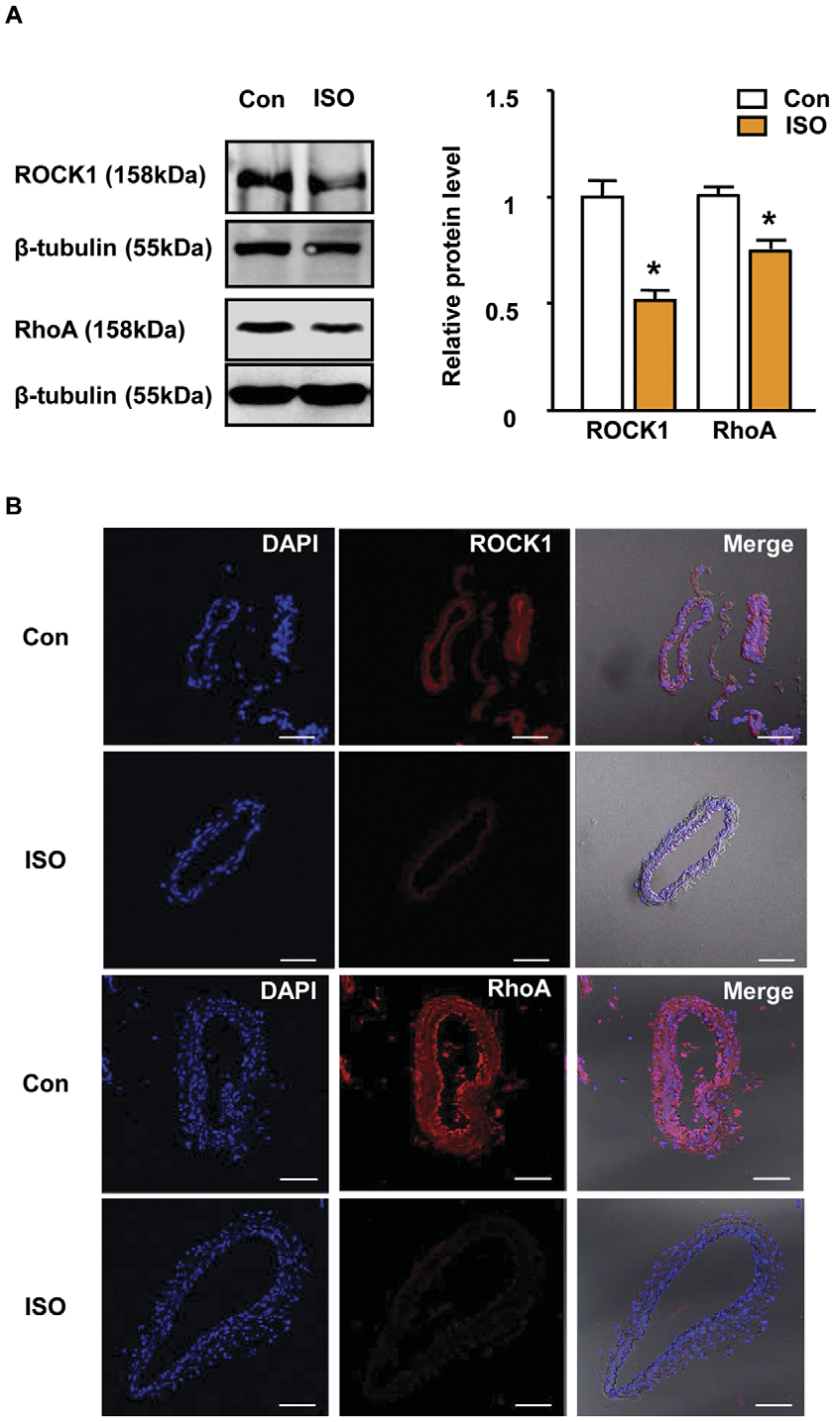
Validation of predicted partner using western blot and IHC. A. Western blot result of ROCK1 and RhoA in control and ISO-CAs (n = 3, mean ± SEM, *p<0.05, two-tailed student t-test). B. Protein expression levels of ROCK1 and RhoA were determined by immunohistochemistry in control and ISO-CAs. Scale bar: 50 μm.

### Lipid peroxidation assay

Lipid peroxidation or malondialdehyde (MDA) assay is a well-established method for measuring oxidative cellular injury in cells and tissues. MDA concentration in Con and ISO-CAs were evaluated using lipid peroxidation assay kit (FR12, Oxford biomedical, USA)[19].

**Figure 5:**
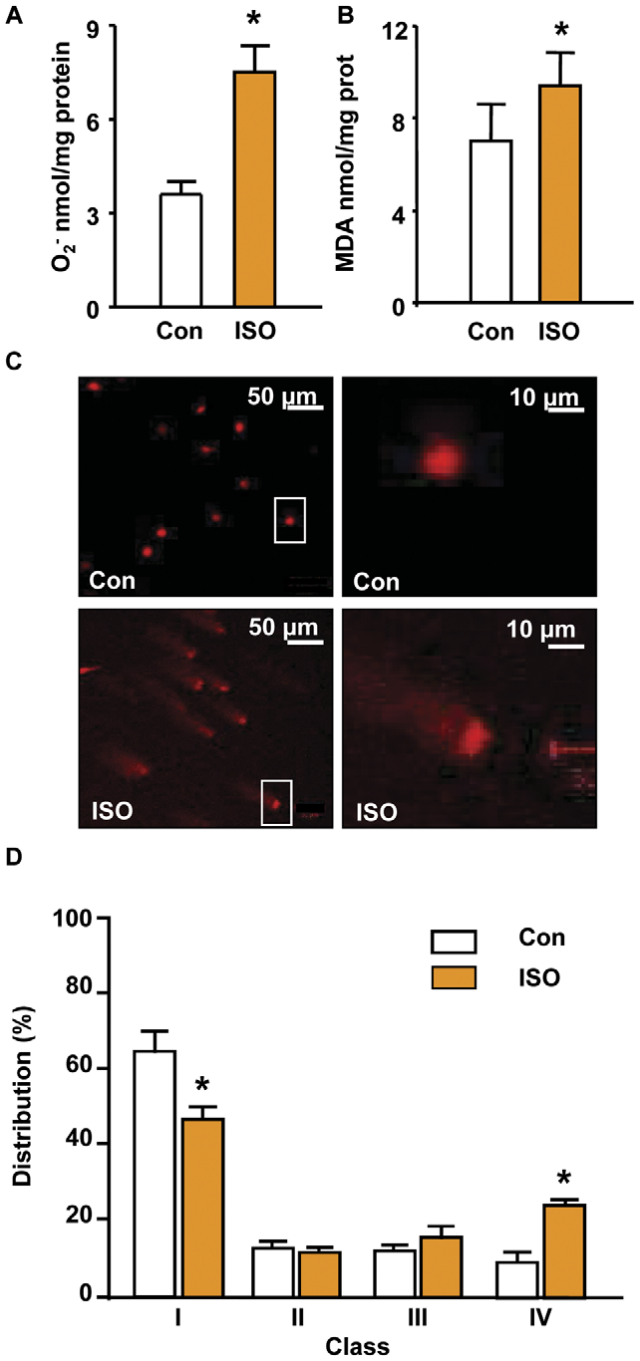
Increased oxidative stress in ISO stimulated cerebral arteries (ISO-CAs). A. NADPH-dependent O2- production was highly increased in ISO-CAs group (7.5±0.85 O_2_- nmol/mg protein) than control (3.6±0.40; mean ± SEM, Student's t-test, * P<0.05 vs. Con, n = 6). B. ROS-induced lipid oxidation was highly increased in ISO-CAs group. C. Oxidative DNA damage in smooth muscle cells (SMCs) from ISO-CAs was assessed by comet assay. Nuclear DNA was stained with propidium iodide and examined under a confocal laser scanning microscope. More highly damaged DNA was observed in SMCs from ISO-CAs (c, d) compared to the control (a, b) based on tail length. Magnification: 100× for a and c, 500× for b and d. D. Statistical summary obtained from comet image analysis. Less damaged cells, class I, were significantly more common in control (65.1±5.9%) than in ISO- (47.5±3.6%) CAs, whereas severely damaged cells, class IV, were markedly more frequent in ISO-CAs (24.7±1.3%) than in the control (9.5±3.2%; mean ± SEM, Student’s t-test, * P<0.05 vs. Con, n = 4).

### DNA damage assessment

To confirm if the alteration of O_2_^-^ production increase the oxidative damage in ISO-CA, oxidative damage levels of DNA in Con (n = 4) and ISO- CA (n = 4) were measured using single-cell gel electrophoresis assay (comet assay) as previously described [20]. Isolated cerebral artery SMCs (n = 4 in each group) were mixed in 300 μl of low-melting point agarose (1% in PBS). Next, 70 μl aliquots were layered onto four agarose-pre-coated slides and cooled at 4°C for 5 min. To eliminate nuclear membranes, proteins, and all non-nuclear components, the embedded cells were lysed overnight in lysis buffer containing 2.5 M NaCl, 0.1 M EDTA, 10 mM Tris, and 1% Triton X-100 (v/v) adjusted to pH 10 with NaOH. Cellular DNA was enzymatically digested with endonuclease III at 37°C for 45 min. Electrophoresis was carried out at 300 mA/25 V for 40 min. After electrophoresis, the samples were washed three times in 250 ml of neutralizing solution and DNA was stained with 20 μl of propidium iodide. Stained DNA samples were observed under a laser-scanning microscope. DNA damage was classified into four categories based on comet tail length (in μm) as follows: class I (0–15), class II (16–30), class III (31–45), and class IV (>46). The frequency of each class was analyzed using LSM510-META software. Roughly 50–60 comets were scored for each slide.

**Figure 6:**
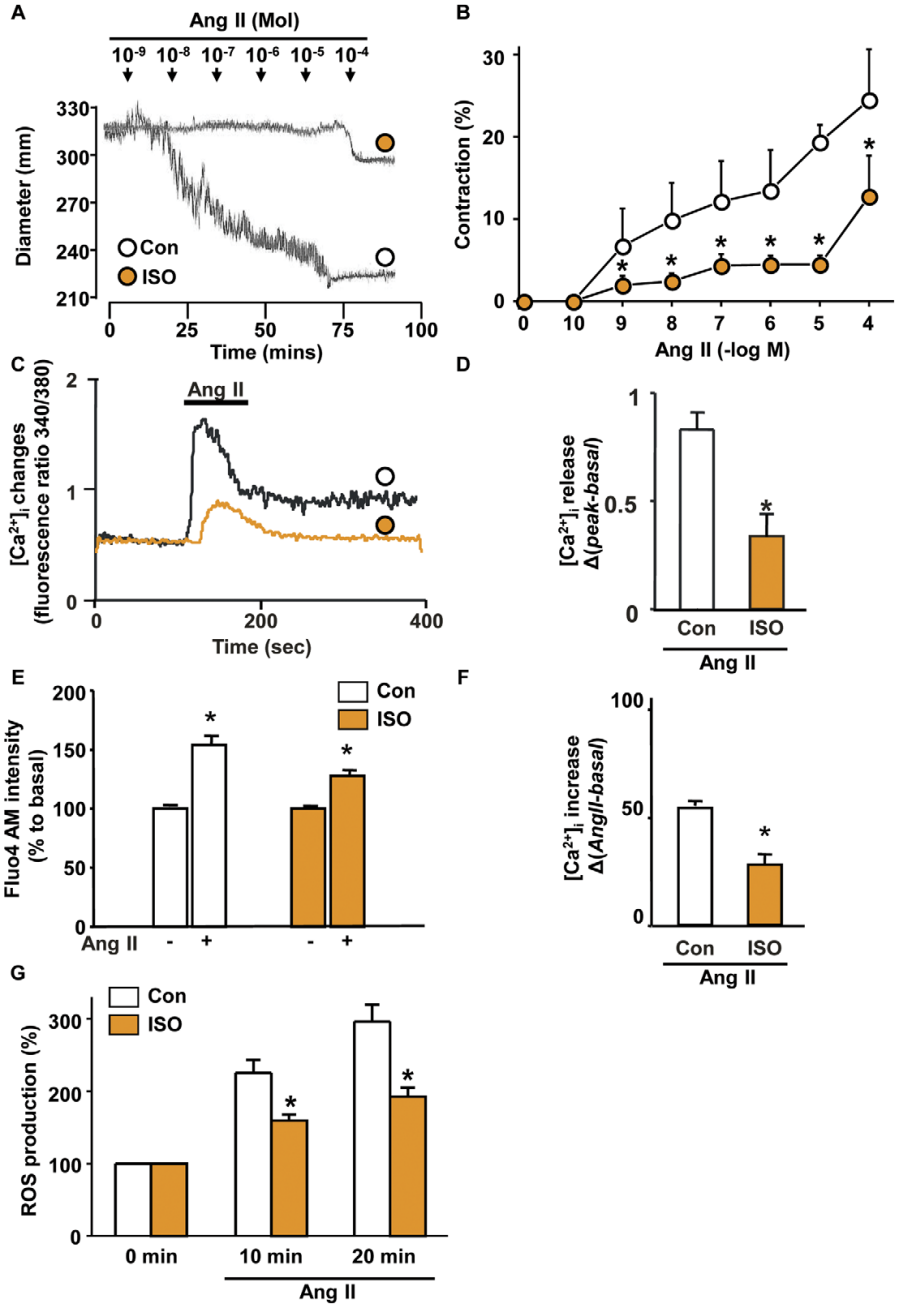
Altered arterial contractility to a vasoconstrictor (Ang II) in ISO-CAs. A. Representative trace of Ang II dose response vascular contraction in control and ISO-CAs. B. Line graph of contractile dose response to Ang II in control and ISO-CAs. (n = 4, mean ± SEM, two-way ANOVA test, *P<0.05). C. Ang II–induced intracellular calcium peak changes in control and ISO-CAs. D. Quantitative group data of calcium release, Ang II-induced [Ca^2+^]_i_ change calculated by Δ(*peak*340/380-*basal*340/380) of each group. (n = 4, mean ± SEM, Student's t-test,*P<0.05). E. F. Intracellular calcium changes were measured by confocal microscopy in basal and after 20 min of Ang II treatment. ISO-CAs has lower response to Ang II-induced intracellular calcium increase (52.35±1.23 vs. 25.76±2.12, n = 4, Student's t-test,*P<0.05). G. Ang II-induced ROS productions were measured by confocal microscopy in basal (0 min) and after 10 and 20 min of Ang II treatment. ISO-CAs has lower response to Ang II-induced ROS production.

### Two-dimensional gel electrophoresis (2-DE) proteome analysis

Proteomic differences between normal control CA and ISO-CA were assessed by 2-DE proteome analysis and MALDI-TOF MS analysis as previously described [21]. Collected normal (n = 3) and ISO-CA (n = 3) were dissolved by repeated vortexing and sonication in ice-cold lysis buffer (7 mol/L urea, 2 mol/L thiourea, 4% CHAPS, 40 mM Tris base, 1% DTT, 0.5% IPG buffer, 0.5% Triton X-114, and protease inhibitor cocktail) for 1 h. The CA protein concentration was assayed using a 2D Quant kit (GE Healthcare) following manufacturer's instruction, and two-dimensional gel electrophoresis (2-DE) and proteome analysis were performed. Each sample was run in duplicate. 13 cm-long dehydrated and immobilized pH gradient (IPG) strips with nonlinear pH range from 3 to 10 were rehydrated overnight in rehydration tray with 250 μl of Destreak^TM^ rehydration solution (GE Healthcare) containing 2% IPG buffer (v/v). Subsequently, 50 μg of the soluble CA proteins in total 100 μl sample solution were loaded by cup loading method. Isoelectric focusing was carried out at 80,000 V/h at 20°C as follows: 500 V for 1 h, 1,000 V for 1 h, and finally gradual voltage increase from 8,000 V to 80,000 V over an hour. After focusing, the IPG strips were placed in 5 ml of an equilibration solution (50 mM Tris-HCl, pH 8.8, containing 6 M urea, 30% glycerol, 2% SDS, and bromophenol blue) that contained 1% DTT (v/v) during the first equilibration step and 2.5% iodoacetamide (v/v) during the second equilibration step (15 min per equilibration step). The 2D separation was performed using the SE600 system (Amersham). The IPG strips were loaded onto a 12.5% SDS-polyacrylamide gel and sealed with low melting point agarose. 4 L running buffer (25 mM Tris, 192 mM glycine, and 3.5 mM SDS at pH 8.3) was filled in the electrophoresis chamber, and 80 volt was applied for 30 min in the first step and 200 volt for around 4 hr in the second step, until the probe dye reached 1 mm distance from the bottom of the gel. The gels were then stained with silver nitrate.

**Figure 7:**
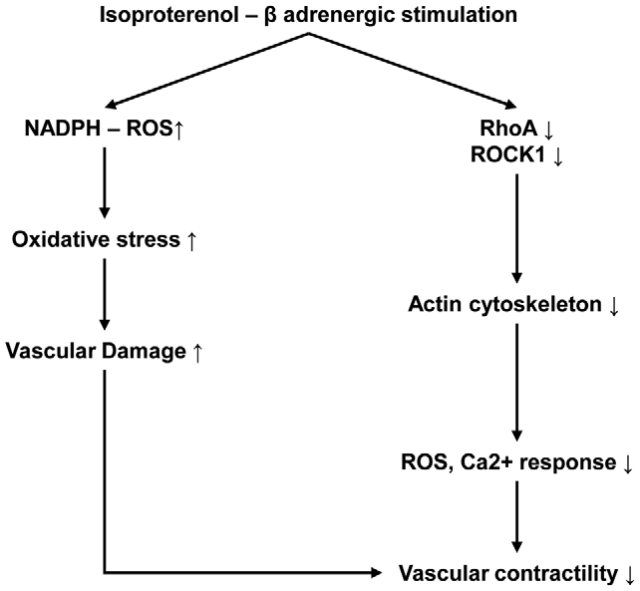
Schematic illustration of β-adrenergic overstimulation-induced vascular dysfunction.

Silver-stained gels were scanned on a flatbed scanner (Umax PowerLook 1100; Fremont, CA, USA), and the digitized images were analyzed using automated image analysis algorithm software (ImageMaster 2D Platinum version 5.0, Amersham Biosciences). Spots showing the same distribution pattern in all gels were selected for further analysis. To increase the confidence level, we filtered the detected protein spots with class gap values over 0 and selected spots that showed a >1.5-fold change in expression and Student's t-test P-value of <0.05 compared to the control. Protein quantification was calculated using the percent volumes (% Vol) of identified proteins for control and isoproterenol stimulated cerebral artery as shown in the equation below.
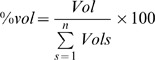
()where Vol_s_ is the volume of spot S in a gel containing n spots.

### Protein identification using MALDI-TOF

Selected spots were enzymatically digested by trypsin and analyzed using matrix-assisted laser desorption ionization time of flight (MALDI-TOF). Selected protein spots were excised from each gel and destained with 30 mM potassium ferricyanide and 100 mM sodium thiosulfate. After washing with 50% acetonitrile (ACN), gel fragments were dried in a vacuum centrifuge. The protein in gel was digested by 0.5 μg of sequencing-grade trypsin (Promega, Southampton, UK) in 20 μl of 25 mM ammonium bicarbonate at 37°C overnight. Digested peptides were extracted with 0.5% trifluoroacetic acid (TFA)/50% ACN solution, and then desalted using ZipTip C18 (Millipore, Bedford, MA) tip. Peptides were eluted directly onto MALDI target by α-cyano-4-hydroxy-cinnamic acid (CHCA) matrix solution (10 mg/ml CHCA in 0.5% TFA/50% acetonitrile (1∶1, (v/v)). All mass spectra were acquired at a reflection mode by a 4700 Proteomics Analyzer (Applied Biosystems, Framingham, MA). The MS spectra were analyzed using Peak Explorer^TM^ 3.0 (Applied Biosystems) software. Resulting data were analyzed by GPS Explorer^TM^ 3.5 (Applied Biosystems) software. The proteins were identified by searching Mammalia of the National Center for Biotechnology Information (NCBI) protein databases using MASCOT 2.0 search algorithm (Matrix Science, London).

### Western blotting

Randomly selected proteome analysis results were confirmed by Western blotting analysis as previously described [22]. Briefly, the protein concentration of each sample (n = 3 in each group) was determined using the bicinchoninic acid (BCA) protein assay. Samples containing 20 μg of total protein were subjected to 12% SDS-polyacrylamide gel electrophoresis (PAGE) and then electrophoretically transferred to a polyvinylidene difluoride (PVDF) membrane. After blocking with 5% non-fat dried milk in Tris-buffered saline/Tween-20 (TBST) for 1 h at room temperature, the membrane was incubated for 2 h with randomly selected primary antibodies including anti-actin related protein (ACTR1A, 43 kDa, 1∶2000 diluted, Abcam, Cambridge, UK), actin related protein 2 (ACTR2, 45 kDa, 1∶2000 diluted, Abcam, Cambridge, UK), α-actin (39 kDa, Abcam, 1∶2000 diluted, Cambridge, UK) , vimentin (45 kDa, Abcam, 1∶1000 diluted, Cambridge, UK), protein phosphatase 1 beta (37 kDa, Abcam, 1∶1000 diluted, Cambridge, UK), NADH dehydrogenase complex subunit 1 (79 kDa, Abcam, 1∶2000 diluted, Cambridge, UK), glutathione-s-transferase (27 kDa, Abcam, 1∶1000 diluted, Cambridge, UK), Ras homolog gene family, member A (RhoA, 24 kDa, 1∶1000 diluted, Santa Cruz Biotechnology, EU) and Rho-associated coiled-coil containing protein kinase 1 (ROCK1, 158 kDa, 1∶1000 diluted, Abcam, Cambridge, UK), respectively. After three washes in TBST, the membrane was incubated for 1 h with a specific peroxidase-coupled secondary antibody. The membrane was washed again, and the signals were visualized using an enhanced chemiluminescence (ECL) image detector (LAS-3000, Fujifilm, Miyagi, Japan). The relative intensities of protein bands were analyzed via densitometry using a gel documentation system and Multi Gauge software (Fujifilm). Finally the target protein band intensity was normalized with intensity of housekeeping protein, β-actin (42 kDa, Abcam, 1∶2000 diluted, Cambridge, UK) or β-tubulin (55 kDa, Abcam, 1∶2000 diluted, Cambridge, UK).

### Immunohistochemistry

Isolated CAs were washed with phosphate buffer saline (PBS) twice to remove blood. CAs were fixed by 4% formaldehyde in PBS for 24 hours. Fixed CAs were rinsed three times by PBS. For making frozen block, they were put into the mold and filled by optimal cutting temperature compound (Sakura Finetek, Torrance, CA, USA). The molds immediately were put into liquid nitrogen. Frozen tissues were sectioned by cryostat microtome (Leica, Nussloch, Germany) for immunohistochemistry. The slides were rinsed three times in PBS in 5 minutes then were blocked by CAS-Block (Invitrogen, Frederick, MD, USA) for 1 hour and then were applied 1∶200 diluted α-actin, ROCK1 and RhoA primary antibody at 4°C for overnight. The slides were rinsed three times by PBS for 10 minutes and then they were applied 1∶100 diluted Alexa Fluor 594 (Invitrogen, Frederick, MD, USA) or FITC (Invitrogen, Frederick, MD, USA) at 37°C for 1 hour. The slides were rinsed three times by PBS for 10 minutes and then they were covered with Prolong® Gold Antifade Reagent with DAPI (Invitrogen, Frederick, MD, USA). Fluorescent images were observed and analyzed under a Zeiss 700 Laser-scanning confocal microscope (Zeiss, Göettingen, Germany).

### Functional annotation and associated network analysis

In order to understand the physiological significance of proteomic alterations in ISO-CAs, we categorized and annotated the identified proteins using orthologous groups of proteins (COGs) algorithm (http://www.ncbi.nlm.nih.gov/COG/grace/fiew.cgi) and **P**rotein **AN**alysis **TH**rough **E**volutionary **R**elationships (PANTHER) classification algorithm (http://pantherbeta.ai.sri.com/help/PANTHERhelp.jsp) [23]. We analyzed protein-protein interaction and functional associations between identified proteins and constructed a functional network using the online STRING 8.0 database (http://string.embl.de) [24]. The constructed protein network was visualized with Cytoscape software (Version 2.6.3).

### Statistical analysis

All results were expressed as mean ± standard error (SE). Differences between control and ISO-CAs were analyzed using two-tailed Student's *t*-test. Significance of dose-dependent Ang II induced vascular contraction between control and ISO-CAs was tested by two-way ANOVA analysis using Origin Pro 8.0. P-values <0.05 were considered statistically significant.

## Results

### Effect of ISO-βAR stimulation on the heart and hemodynamics

After 7 days of daily ISO injection, hearts of ISO treated animals were significantly enlarged than normal group animals (Figure S1A). The heart weight and heart to body weight ratio of ISO group were also significantly increased than those of normal group (Figure S1B) indicating successful ISO-βAR stimulation in the model animal. However, systolic, diastolic, mean arterial pressure and heart beat rate were not significantly altered by ISO treatment (Table 1) indicating there was no hemodynamic effect of ISO-βAR stimulation on cerebrovasculature. Unlike to hypertrophied heart, there is no significant alteration of arterial lumen and thickness between control and ISO treated cerebral arteries (Figure S1C). In addition, the ISO injection did not alter expression of angiotensin II type 1 (AT1R) and type 2 (AT2R) receptors itself (Figure S1D).

### Proteomic alteration in ISO-CAs

Protein spots (981.0±61.0) were detected from scanned 2-DE gel images. Major spot dispersions were observed over a pH range of 4–9 and a molecular weight range of 10–100 kDa. The expression patterns of CA proteins in normal and ISO- rabbits are shown in Figure 1A. We identified differentially expressed 32 proteins including 2 up- and 30 down-regulated proteins using MALDI-TOF MS analysis (Figure S2). Detailed MS information of identified proteins was listed in supporting information Table S1 and Figures S3 and S4. The identified proteins were divided into 11 groups based on COG category and their expressional changes are listed in Table 2. Through annotation and categorization of identified proteins, we found that modulated proteins were widely associated with regulation of cytoskeletons (Figure 1C and D). Specifically, the expression of actin gamma 2 (ACTA3, 54%), actin-related protein 1 (ACTR1A, 50%), actin-related protein 2 (ACTR2, 47%), α-actin (ACTC1, 37%), capping protein muscle Z-line (CAPZB, 55%), vimentin (VIM, 36%) and serine/threonine-protein phosphatase PP1-beta catalytic subunit (PPP-1B) decreased significantly in ISO-CAs (Figure 1B). Additionally, expression of several neuroprotective chaperones and protein maturation elements, such as heat shock protein 9A (mortalin, 42%) and stress-induced-phosphoprotein 1 (STI1, 55%) were markedly down-regulated in ISO-CAs (Table 2 and Figure 1B).

### Validation of Proteomic result

Western blotting was used to validate the changes in the expression of cytoskeletal proteins, which are closely related to CA contraction and dilation. The expression of cytoskeletal components and regulatory proteins, including the ACTR1A, ACTR2, α-actin, VIM and PPP-1B, significantly decreased in the ISO-CAs. In addition, the protein expression levels of NADH dehydrogenase and glutathione-S-transferase were confirmed to be increased in the ISO-CAs (Figure 2A and B). Decreased α–actin level was further validated in ISO-CAs by immunohistochemistry (Figure 2C).

### Functional association of altered proteins

In the primary protein-protein interaction search, we constructed three discontinuous clusters consisting of 23 protein nodes with 26 interactions among the nodes; the 9 identified proteins did not have known direct interaction with others (Figure 3A). Subsequently, to extend the interacting partner search, we found 16 predicted interaction proteins that linked with 23 primary protein nodes, which included two important actin cytoskeleton regulatory proteins, RhoA and ROCK1. Furthermore, protein nodes in the network were marked in four different colors based on their major cellular functions: cytoskeletal and contractile regulation, energy production and conversion, chaperone and cellular signaling, and mitochondrial electron transfer chain. Consequently, we constructed a simple, non-redundant protein network consisting of 48 protein nodes with 94 interactions that may be helpful in understanding proteome-based systemic changes of CA in ISO. Western blotting and immunohistochemistry confirmed the decreased expression of RhoA and ROCK1 in ISO-CAs (Figure 4A and B).

### Superoxide production and DNA damage

To evaluate if ISO-βAR stimulation induces oxidative damage in cerebrovasculature . We measured ROS production, lipid peroxidation and DNA damage in the cerebral arterial SMCs of both group. In the presence of NADPH, O_2_^-^ production was significantly increased in the ISO-CAs group (7.5±0.85 O_2_- nmol/mg protein) than in the control group (3.6±0.40 O_2_- nmol/mg protein, n = 3, p<0.05) (Figure 5A). Increased O_2_^-^ production led directly to lipid oxidation (Figure 5B) and severe DNA damage in ISO-CAs (Figure 5C). Cells containing severely damaged DNA (class IV) were significantly more frequent in the ISO group (24.7±1.3%) than in the control group (9.5±3.2%) (Figure 5D).

### Ang II-induced calcium release and arterial contractility alteration

Ang II-induced vascular contraction experiments were applied to test whether proteomic alterations actually lead to modification of vascular contractile response. As a result of the experiments, high K^+^ and Ang II–induced vascular contraction was significantly impaired in ISO-CAs compared to the control (Figure 6A and B, Figure S5A). As for the causes of contractile dysfunction, we found that ISO treatment significantly reduced Ang II–induced [Ca^2+^]_i_ transient peak (Figure 6C and D) and prolonged [Ca^2+^]_i_ elevation (Figure 6E and F and Figure S5B) and ROS generation rate (Figure 6G and Figure S5C).

## Discussion

Maintenance of normal blood circulation in the cerebral and cardiovascular systems is essential for life. Distorted cerebral homeostasis may aggravate the risk of many life-threatening neurodegenerative events including stroke. The βAR overstimulation-induced cardiac hypertrophy is believed to potentiate cerebral damage even in the absence of clinical symptoms like as hypertension [25], [26], [27], [28], [29], [30], suggesting βAR overstimulation may cause cerebrovascular damage. In our model, ISO injection-induced cardiac hypertrophy without changes of LV systolic pressure and LV end-diastolic pressure indicated successful induction of βAR overstimulation in the model animal. Also, we were able to exclude hemodynamic effect on cerebral artery in the model (Figure S1 and Table 1) [15], [31], [32]. The ISO injection did not alter expression of angiotensin II type 1 (AT1R) and type 2 (AT2R) receptors (Figure S1D).

### Remodeled cytoskeletal proteome network in ISO-CAs

As a core finding of this study, we discovered that ISO significantly remodeled cytoskeletal proteome network, which disrupts vascular responses to Ang II. Major proteomic alteration in ISO-CAs is shown by remarkable down-regulation of cytoskeletal proteins (Table. 2 and Figure 2). This result was in agreement with our previous study that PKA activity and Ras/Raf expressions were significantly decreased in ISO-CAs [4]. Because Ras/Raf/ERK signaling cascade is essential for actin-base cytoskeleton organization, decreased level of those proteins in ISO-CAs interrupted actin cytoskeleton network [11], [12], [13]. As a reliable cause of actin-cytoskeletal disorganization, we found a decreased protein expression level of RhoA and ROCK1 (Figure 4). RhoA and ROCK1 play an essential role in the actin-cytoskeleton organization and smooth muscle contraction via phosphorylation of myosin light chain [33], [34]. Thus, our result suggested that ISO stimulation increased ERK activation [4], [5], which oppositely suppressed ROCK1 activity [35], [36] and led to disorganization of actin-cytoskeleton. In addition, decreased level of vimentin [37] and moesin [38] may be implicated with altered activation of ERK and ROCK1 in ISO-CAs.

Cytoskeleton structure and its components are fundamentally important for maintaining cell shape and integrity. Increasing number of experiments suggest their dynamic role in various biological processes [39], [40], [41]. In the vascular system, contractility of SMCs is widely regulated by cytoskeletal proteome network [42]. Since actin is the major component of this cytoskeletal network, down-regulation of α-actin and actin-related proteins could lead to dysregulation of cytoskeletal network organization of ISO-CAs. Actin is the most essential and fundamental protein unit in terms of cellular structure and has great functional significance in the regulation of contractility in various tissue and muscles. In CAs, dynamic actin cytoskeleton regulates arterial diameter in response to changes in intravascular pressure through the polymerization of monomeric globular (G-) actin into filamentous (F-) actin [40]. Polymerization of the actin cytoskeleton is regulated by the Wiskott−Aldrich syndrome protein (WASP) and the Arp2/3 complex [43]. Furthermore, WASP and Arp2/3 activities are implicated with activities of moesin [44] and coronin 1B [45]. Therefore, down-regulated expression of α-actin, ACTR2 (or Arp2), moesin and coronin 1B suggests that ISO-stimulation could disrupt actin-based polymerization and cytoskeleton organization in CAs.

To evaluate functional relevance of those actin cytoskeletal protein modifications, we investigated the Ang-II-induced Ca^2+^ regulation and contractile response because Ang- II is the most well known vasoconstrictor. Ang II-induced ROS generation and ERK signaling has important role in the Ang II-induced vascular contraction [46], [47]. Actin cytoskeletal network also plays an important role in regulating Ang II-induced Ca^2+^ release from internal stores and Ca^2+^ influx [41] and L-type Ca^2+^ channel current [48]. In agreement with these studies, we found that the disruption of cytoskeletal network in ISO-CAs led to decreased Ang II-induced cellular Ca^2+^ elevation and contraction (Figure 5). As well as in Ca^2+^ signaling, actin-cytoskeleton plays a pivotal role in Ang II-induced ROS generation, which is essential for Ang II-induced vascular contraction [47], [49]. Disruption of actin cytoskeleton using cytochalasin B significantly reduced Ang II-induced ROS generation [49]. Similarly, Ang II-induced ROS generation was significantly decreased with disruption of actin-cytoskeleton in our result (Figure 6G). These results suggest that disruption of actin cytoskeletal network interrupts Ang-II mediated intracellular Ca^2+^ homeostasis, ROS generation and vascular contraction in ISO-CAs.

### Increased oxidative stress in ISO-CA with loss of antioxidative proteins

Although ISO treatment blunted Ang II-ROS generation rate, it increased the basal ROS level and oxidative stress (Figure 5). Similarly, several recent findings demonstrated that ISO stimulation increased reactive oxygen species (ROS) production through βAR in HEK293 cell [50], rat cardiac myocyte [51], rat aorta [15] and DNA damage of rat cardiac myocyte [52]. Our results showed the sensitivity of ISO-CAs to DNA damage. The evidence for accelerated oxidative stress was provided by our proteomic analysis result, which showed significantly decreased expression of several cytoprotective chaperones and protein maturation elements. In particular, decreased levels of HSP9A (mortalin) and stress-induced-phosphoprotein 1(STI1), which are cytoprotective proteins, are deleterious to the cellular anti-oxidative mechanism.

Mortalin is a member of the essential mitochondrial chaperone (heat shock protein 70) family and has a cellular protective role against various oxidative stresses through suppression of ROS production [53] and accumulation [54]. Even though the mechanism through which mortalin suppresses ROS production is unclear, mortalin may stabilize cytochrome c and other components of the electron transport chain (ETC) and thereby suppress mitochondrial ROS production [53]. The STI1, also known as Hsp70/Hsp90-organizing protein (HOP), is a linker of Hsp70 and Hsp90 and regulator of linked chaperones activities [55]. Recent study demonstrated that it has a role in neuroprotection [56] and regulates the activity of superoxide dismutase for ROS scavenging [57]. Thus, down-regulated expression of mortalin and STI1 may cause deleterious oxidative stress on DNA in ISO-CAs. Beside that, Hsp90, which is regulated by HOP, is essential for binding of Raf-1-Ras complex and regulating their activities. Down-regulation of HOP is therefore a significant factor in decreased Ras/Raf activities in ISO-CAs [58].

In addition to biological results, systemic analysis of proteomic data helped us to understand integrative biological significance of each altered proteins and to comprehend complicated interactions among the proteins. Since proteins rarely act alone but rather act in concert with other proteins to constitute a biological pathway [59], it is important to analyze interaction and functional clustering of each altered proteins in ISO-CAs for understanding cerebral arterial dysfunction in βAR overstimulation.

## Conclusion

The present study demonstrated that βAR overstimulation increased oxidative stress by damaging anti-oxidative proteins and impaired contractile response of CA. As a possible mechanism of this abnormality, our results suggested that βAR overstimulation disrupted actin cytoskeleton proteome network through down-regulation of RhoA/ROCK1 proteins and increased oxidative damage, which consequently led to contractile dysfunction in CA (Figure 7). Possible involvement of cytoskeletal disorganization in cerebrovascular dysfunction may give a new insight into understanding cerebral damage after βAR overstimulation and therapeutic intervention of βAR overstimulation induced cerebrovascular damage.

## Supporting Information

Figure S1 The effect of beta adrenergic (βAR) overstimulation in heart and angiotensin 2 receptors in cerebral artery. A. Images of longitudinal sectioned heart of control (left) and isoproterenol injected (right) rabbit. B. Comparative histogram of body weight (BW), heart weight (HW) and HW/BW of control and isoproterenol treated rabbits. C. Images of Hematoxylin and Eosin stained cerebral arteries from control and ISO treated rabbits. D. Western blot analysis of angiotensin 2 type 1 (AT1R) and type 2 (AT2R) receptors in control and ISO-CAs (n = 5 in each group, Student's t-test *P<0.05 vs. control).
(DOC)

Figure S2 ISO-βAR overstimulation-induced proteome changes in cerebral arteries (CAs) identified by comparative 2-DE. Enlarged 2-DE spot images show the alteration of CAs protein expression for each group (C, control; ISO, ISO-βAR overstimulation). Regions of 2-DE gels with reproducible protein alterations are indicated in the box. Significant changes relative to the control proteins are indicated in the graphs to the right of the spot images (*P<0.05, n = 3). Results are categorized under functional clusters of orthologous groups of proteins (COGs).
(DOC)

Figure S3 Down-regulation of anti-oxidative proteins in ISO-βAR overstimulated cerebral artery. Representative set images of gel spot, 3D and MALDI-TOF MS spectra show down-regulation of mortalin ( = heat shock protein 9A, HSP9A)(A) and stress induced phosphoprotein 1A (STIP1) (B) in ISO-CAs.
(DOC)

Figure S4 Down-regulated actin cytoskeletal proteins in ISO-CA. Representative set images of gel spot, 3D and MALDI-TOF MS spectra show down-regulation of α-actin (A), actin related protein 1A (ACTR1) (B) and actin related protein 2 (ACTR2) (C) in ISO-CAs.
(DOC)

Figure S5 A. High K^+^-induced vascular contraction measurement. B. Ang II-induced intracellular Ca^2+^ changes in Con and ISO-CAs. C. Ang II-induced ROS changes in Con and ISO-CAs.
(DOC)

Table S1 The list of identified proteins.
(DOC)
