# Supplementary material for: Beta Adrenergic Overstimulation Impaired Vascular Contractility via Actin-Cytoskeleton Disorganization in Rabbit Cerebral Artery
Source: PLoS One. 2012 Aug 20;7(8):e43884. doi: 10.1371/journal.pone.0043884 (PMC3423383; doi:10.1371/journal.pone.0043884)
Supplement: Figure S1 — The effect of beta adrenergic (βAR) overstimulation in heart and angiotensin 2 receptors in cerebral artery. A. Images of longitudinal sectioned heart of control (left) and isoproterenol injected (right) rabbit. B. Comparative histogram of body weight (BW), heart weight (HW) and HW/BW of control and isoproterenol treated rabbits. C. Images of Hematoxylin and Eosin stained cerebral arteries from control and ISO treated rabbits. D. Western blot analysis of angiotensin 2 type 1 (AT1R) and type 2 (AT2R) receptors in control and ISO-CAs (n = 5 in each group, Student's t-test *P<0.05 vs. control). (DOC) [file pone.0043884.s001.doc]

**
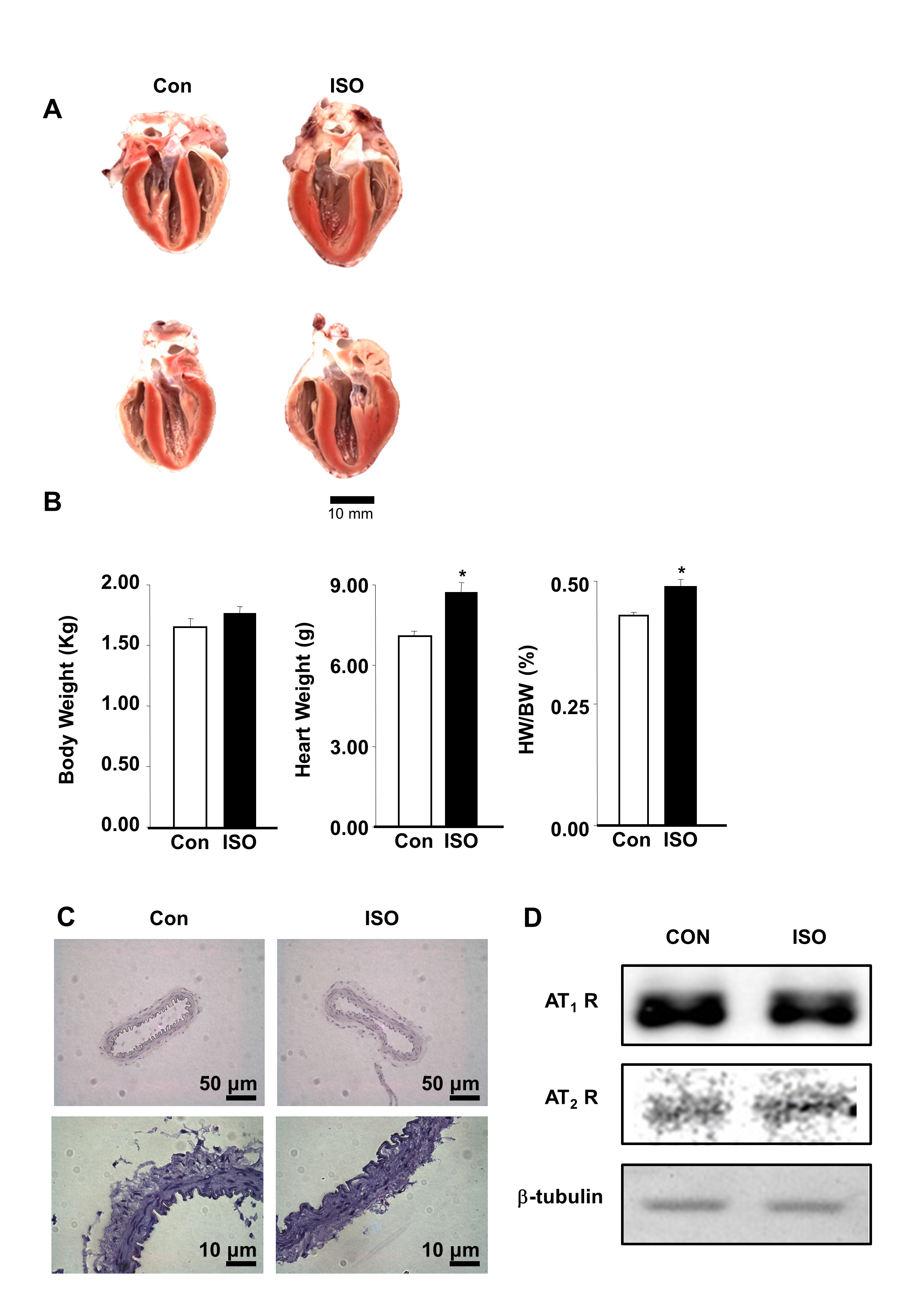
**

Figure S1. The effect of beta adrenergic (βAR) overstimulation in heart and angiotensin 2 receptors in cerebral artery. A. Images of longitudinal sectioned heart of control (left) and isoproterenol injected (right) rabbit. B. Comparative histogram of body weight (BW), heart weight (HW) and HW/BW of control and isoproterenol treated rabbits. C. Images of Hematoxylin and Eosin stained cerebral arteries from control and ISO treated rabbits. D. Western blot analysis of angiotensin 2 type 1 (AT1R) and type 2 (AT2R) receptors in control and ISO-CAs (n= 5 in each group, Student’s t-test *P<0.05 vs. control).
