# Supplementary material for: Beta Adrenergic Overstimulation Impaired Vascular Contractility via Actin-Cytoskeleton Disorganization in Rabbit Cerebral Artery
Source: PLoS One. 2012 Aug 20;7(8):e43884. doi: 10.1371/journal.pone.0043884 (PMC3423383; doi:10.1371/journal.pone.0043884)
Supplement: Figure S2 — ISO-βAR overstimulation-induced proteome changes in cerebral arteries (CAs) identified by comparative 2-DE. Enlarged 2-DE spot images show the alteration of CAs protein expression for each group (C, control; ISO, ISO-βAR overstimulation). Regions of 2-DE gels with reproducible protein alterations are indicated in the box. Significant changes relative to the control proteins are indicated in the graphs to the right of the spot images (*P<0.05, n = 3). Results are categorized under functional clusters of orthologous groups of proteins (COGs). (DOC) [file pone.0043884.s002.doc]

**
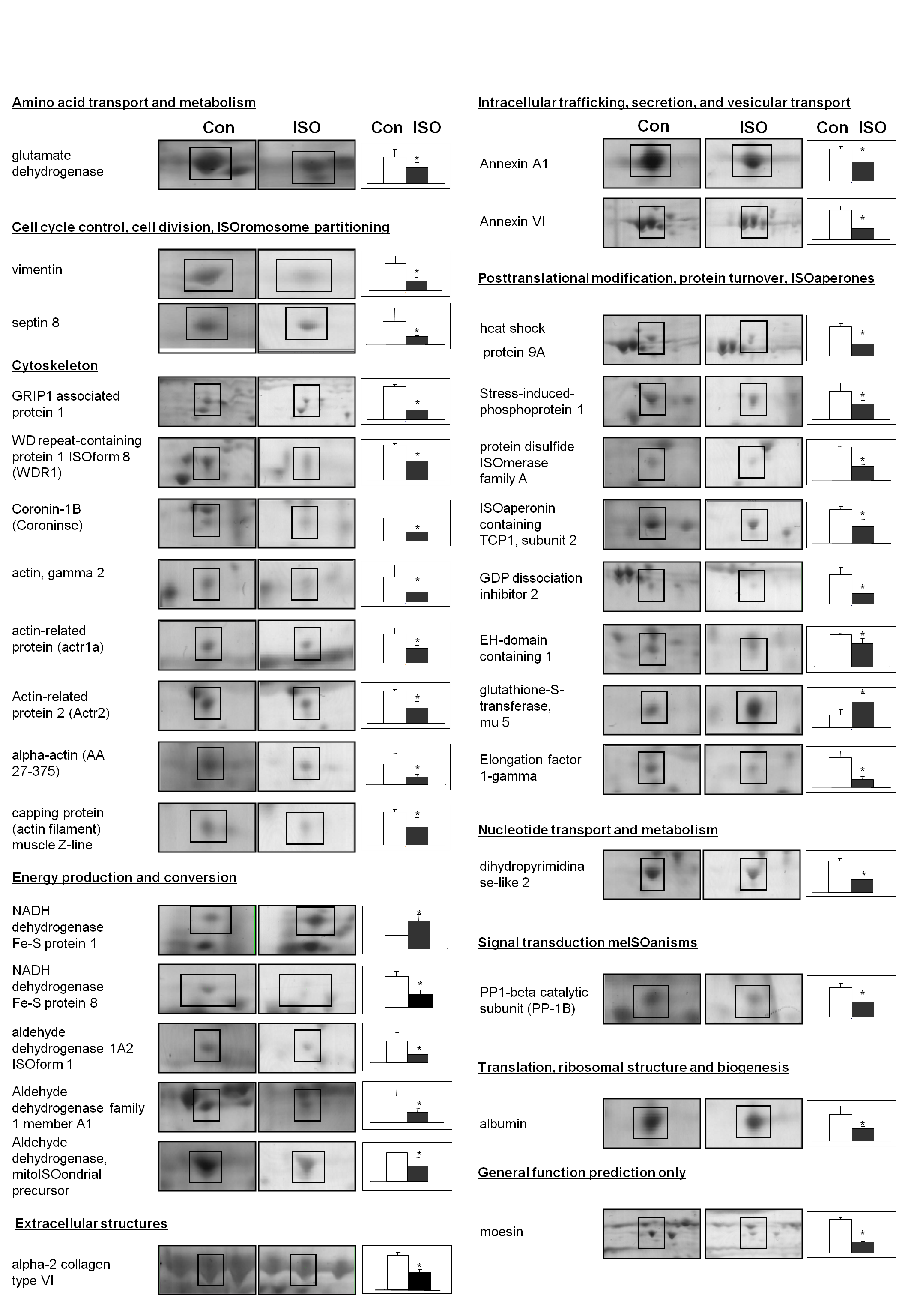
**

Figure S2. ISO-βAR overstimulation-induced proteome changes in cerebral arteries (CAs) identified by comparative 2-DE. Enlarged 2-DE spot images show the alteration of CAs protein expression for each group (C, control; ISO, ISO-βAR overstimulation). Regions of 2-DE gels with reproducible protein alterations are indicated in the box. Significant changes relative to the control proteins are indicated in the graphs to the right of the spot images (*P < 0.05, n = 3). Results are categorized under functional clusters of orthologous groups of proteins (COGs).
