# Supplementary material for: Beta Adrenergic Overstimulation Impaired Vascular Contractility via Actin-Cytoskeleton Disorganization in Rabbit Cerebral Artery
Source: PLoS One. 2012 Aug 20;7(8):e43884. doi: 10.1371/journal.pone.0043884 (PMC3423383; doi:10.1371/journal.pone.0043884)
Supplement: Figure S3 — Down-regulation of anti-oxidative proteins in ISO-βAR overstimulated cerebral artery. Representative set images of gel spot, 3D and MALDI-TOF MS spectra show down-regulation of mortalin ( = heat shock protein 9A, HSP9A)(A) and stress induced phosphoprotein 1A (STIP1) (B) in ISO-CAs. (DOC) [file pone.0043884.s003.doc]

**
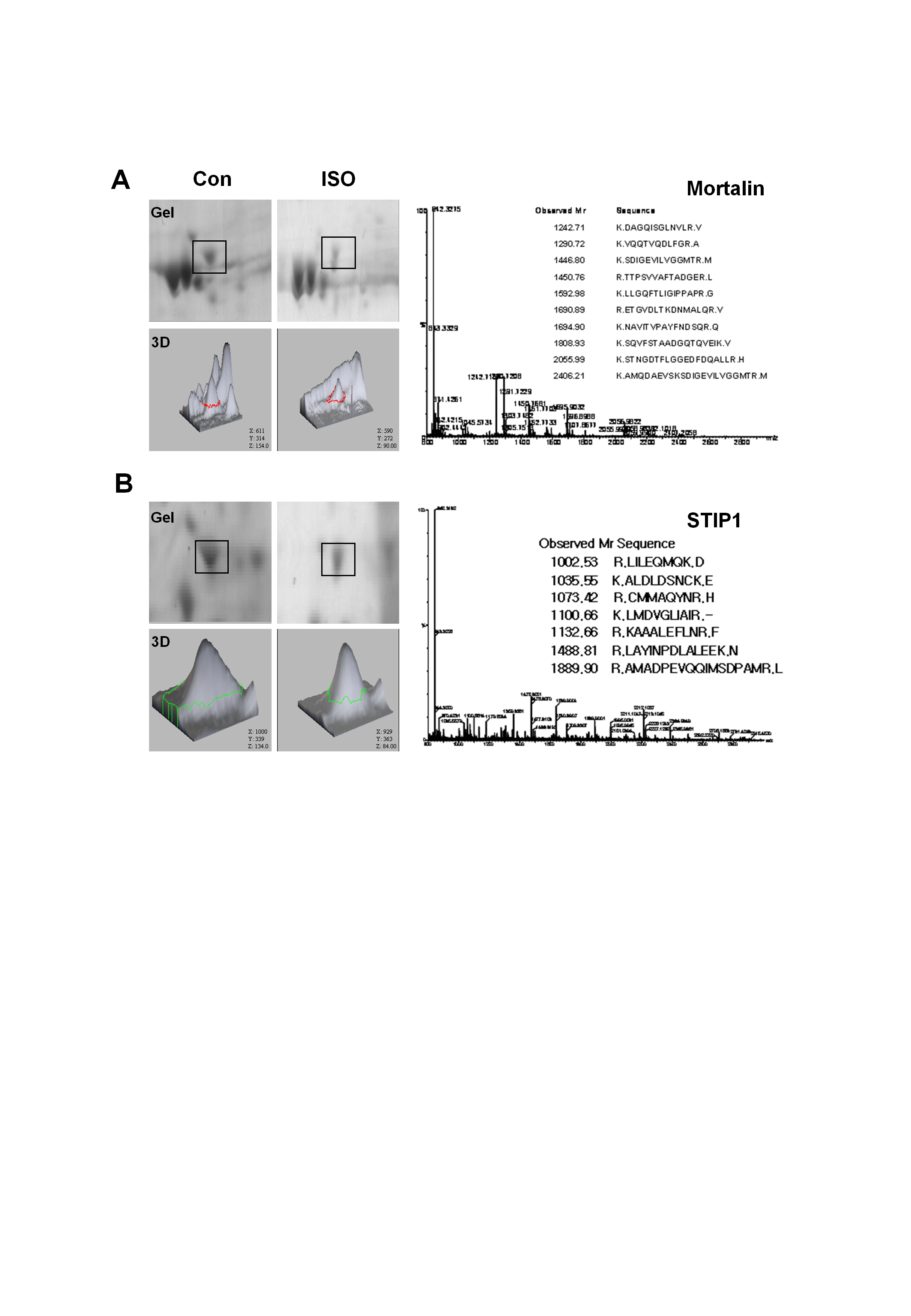
** Figure S3. Down-regulation of anti-oxidative proteins in ISO-βAR overstimulated cerebral artery. Representative set images of gel spot, 3D and MALDI-TOF MS spectra show down-regulation of mortalin (=heat shock protein 9A, HSP9A)(**A**) and stress induced phosphoprotein 1A (STIP1) (**B**) in ISO-CAs.
