# Supplementary material for: Beta Adrenergic Overstimulation Impaired Vascular Contractility via Actin-Cytoskeleton Disorganization in Rabbit Cerebral Artery
Source: PLoS One. 2012 Aug 20;7(8):e43884. doi: 10.1371/journal.pone.0043884 (PMC3423383; doi:10.1371/journal.pone.0043884)
Supplement: Figure S4 — Down-regulated actin cytoskeletal proteins in ISO-CA. Representative set images of gel spot, 3D and MALDI-TOF MS spectra show down-regulation of α-actin (A), actin related protein 1A (ACTR1) (B) and actin related protein 2 (ACTR2) (C) in ISO-CAs. (DOC) [file pone.0043884.s004.doc]

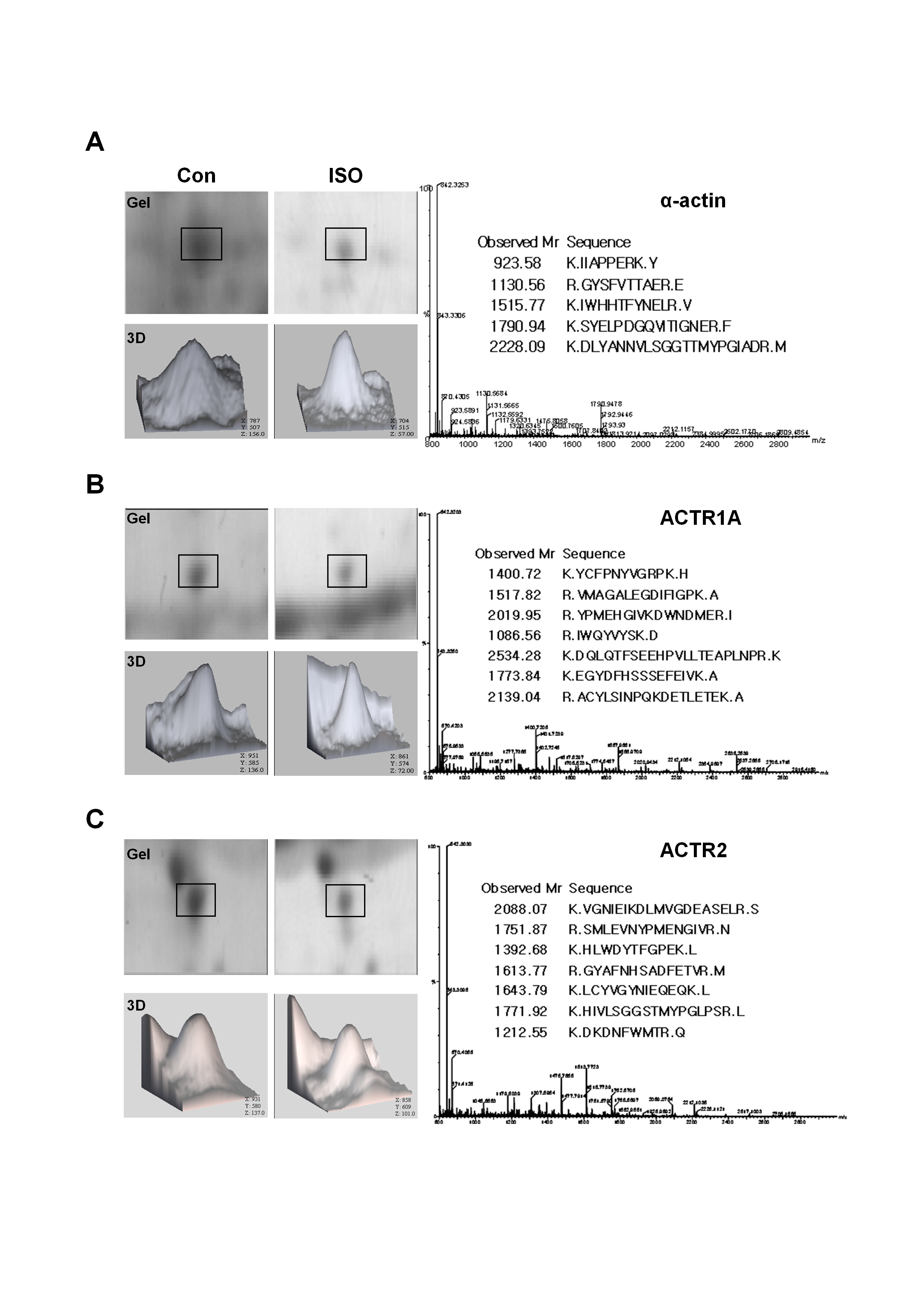
Figure S4. Down-regulated actin cytoskeletal proteins in ISO-CA. Representative set images of gel spot, 3D and MALDI-TOF MS spectra show down-regulation of α-actin (**A**), actin related protein 1A (ACTR1) (**B**) and actin related protein 2 (ACTR2) (**C**) in ISO-CAs
