# Supplementary material for: Beta Adrenergic Overstimulation Impaired Vascular Contractility via Actin-Cytoskeleton Disorganization in Rabbit Cerebral Artery
Source: PLoS One. 2012 Aug 20;7(8):e43884. doi: 10.1371/journal.pone.0043884 (PMC3423383; doi:10.1371/journal.pone.0043884)
Supplement: Figure S5 — A. High K+-induced vascular contraction measurement. B. Ang II-induced intracellular Ca2+ changes in Con and ISO-CAs. C. Ang II-induced ROS changes in Con and ISO-CAs. (DOC) [file pone.0043884.s005.doc]

**
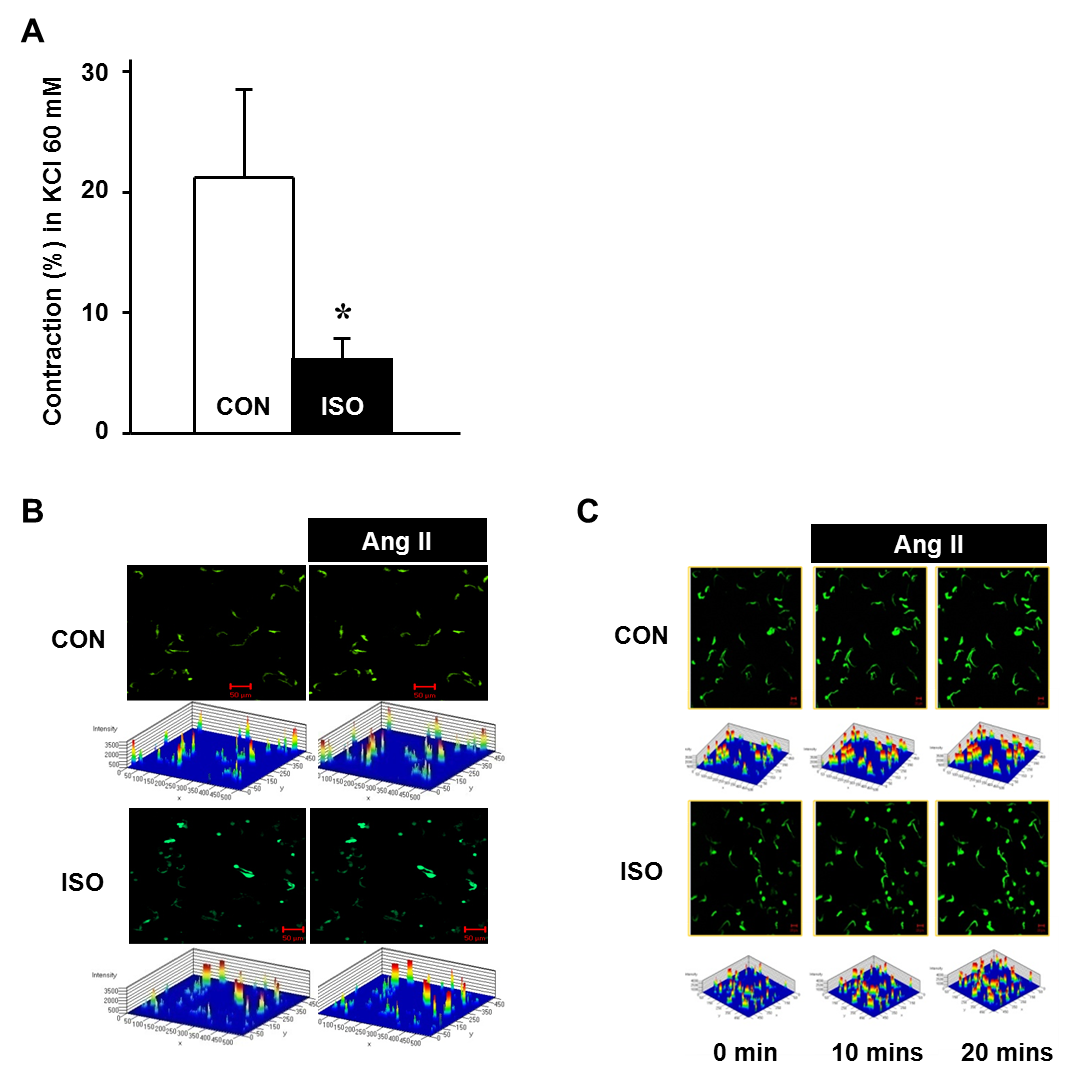
**

Figure S5. A. High K^+^-induced vascular contraction measurement. B. Ang II-induced intracellular Ca^2+^ changes in Con and ISO-CAs. C. Ang II-induced ROS changes in Con and ISO-CAs.
