# Supplementary material for: Beta Adrenergic Overstimulation Impaired Vascular Contractility via Actin-Cytoskeleton Disorganization in Rabbit Cerebral Artery
Source: PLoS One. 2012 Aug 20;7(8):e43884. doi: 10.1371/journal.pone.0043884 (PMC3423383; doi:10.1371/journal.pone.0043884)
Supplement: Table S1 — The list of identified proteins. (DOC) [file pone.0043884.s006.doc]

**Table S**1. The list of identified proteins.

| **Spot** | **GI^1^** | **Identification** | **Gene** | **Mw^2^** | **Pi^3^** | **Score^4^** | **Pep^5^** | **Cov^6^.** | **Pep. Mr^7^** | **Matched peptide sequences** |
| --- | --- | --- | --- | --- | --- | --- | --- | --- | --- | --- |
| **No.** |  |  |  | **(kd)** |  |  |  | **(%)** |  |  |
| **1,2** | **49907** | **alpha-2 collagen type VI** | **COL6A2** | **81** | **6.36** | **31** | **1** | **2*** | **1742.8991** | **K.NLEWIAGGTWTPSALK.F** |
| **17** | **53850628** | **NADH dehydrogenase (ubiquinone) Fe-S protein1** | **NDUFS1** | **80** | **5.65** | **130** | **11** | **22** | **1160.5826 1607.7791 2120.0783 1901.8577  968.5079  1011.5138 1402.7820 2070.1472 1504.8071 2074.0946 2118.9381** | **K.GWNILTNSEK.S  R.FASEIAGVDDLGTTGR.G  R.KTESIDVMDAVGSNIVVSTR.T R.MHEDINEEWISDKTR.F  R.FAYDGLKR.Q  K.SWLHNDLK.V  K.VALIGSPVDLTYR.Y  R.IASQVAALDLGYKPGVEAIR.K  K.LLFLLGADGGCITR.Q  R.ALSEIAGITLPYDTLDQVR.N  R.YDDVEEANYFQQASELAK.L** |
| **18** | **6754256** | **heat shock protein 9A** | **HSPA9** | **74** | **5.91** | **128** | **10** | **20** | **1693.8423  1241.6727  2054.9545  1689.8355  1449.7517  2405.1929  1445.7547  1289.6728  1807.8952  1591.9449** | **K.NAVITVPAYFNDSQR.Q  K.DAGQISGLNVLR.V  K.STNGDTFLGGEDFDQALLR.H  R.ETGVDLTKDNMALQR.V  R.FVSWFLGHSSRK.A  K.AMQDAEVSKSDIGEVILVGGMTR.M  K.SDIGEVILVGGMTR.M  K.VQQTVQDLFGR.A  K.SQVFSTAADGQTQVEIK.V  K.LLGQFTLIGIPPAPR.G** |
| **19,20** | **13540689** | **Moesin** | **MSN** | **68** | **6.16** | **120** | **14** | **18** | **946.5270  2065.0037  2080.9986  975.5389   893.5374  1232.6050  1535.8017  1551.7966  1103.5763   958.5851  1086.6801  1309.6819  1181.5869  1471.7817** | **-.MPKTISVR.V Oxidation (M)  R.VTTMDAELEFAIQPNTTGK.Q  R.VTTMDAELEFAIQPNTTGK.Q Oxidation (M)  K.QLFDQVVK.T  R.LFFLQVK.E  R.IQVWHEEHR.G  R.GMLREDAVLEYLK.I  R.GMLREDAVLEYLK.I Oxidation (M)  K.IGFPWSEIR.N  K.FVIKPIDK.K  K.FVIKPIDKK.A  K.KAPDFVFYAPR.L  K.APDFVFYAPR.L  R.RKPDTIEVQQMK.A** |
| **21** | **71773329** | **annexin VI isoform 1** | **ANXA6** | **76** | **5.42** | **121** | **13** | **18** | **1073.5903  1781.8736  1025.5433  1767.9003  1700.8629   998.6124  1479.6994  1620.7379  1040.5147  1056.5096  1065.5971   909.4960  1091.6121** | **K.CLIEILASR.T  K.WGTDEAQFIYILGNR.S  R.LVFDEYLK.T  K.GLGTDEDTIIDIITHR.S  R.LILGLMMPPAHYDAK.Q 2 Oxidation (M)  K.ALIEILATR.T  R.AINEAYKEDYHK.S  K.SLEDALSSDTSGHFR.R  R.FMTILCTR.S  R.FMTILCTR.S Oxidation (M)  R.RVFQEFIK.M  R.VFQEFIK.M  K.TLTRIMVSR.S Oxidation (M)** |
| **24** | **40254781** | **GDP dissociation inhibitor 2** | **GDI2** | **51** | **5.93** | **86** | **9** | **23** | **937.4869  1124.5900  1311.6935  1492.7344  1605.6803  1621.6752  1898.7775  2140.0992  2198.0498** | **K.LYSESLAR.Y  K.MLLFTEVTR.Y Oxidation (M)  R.GRDWNVDLIPK.F  K.QLICDPSYVKDR.V  R.MTGSEFDFEEMKR.K  R.MTGSEFDFEEMKR.K Oxidation (M)  R.TDDYLDQPCCETINR.I  K.SPYLYPLYGLGELPQGFAR.L  K.NTNDANSCQIIIPQNQVNR.K** |
| **26** | **74196422** | **GRIP1 associated protein 1** | **GRIPAP1** | **55** | **5.13** | **75** | **8** | **19** | **1437.6558  1101.6142  931.4545  914.5297  1811.9377  1349.7163  2129.0496  925.4505  1272.6673** | **-.MAQALSEEEFQR.M  R.KNGVELSSLR.Q  K.NMAALQER.Y  K.AQLARTQK.L  K.LQQELEAANQSLAELR.D  R.QGERLEHAAALR.A  K.VMLDELAMETLQEKSQHK.E  R.EEHAAELK.G  K.DTVDGQRILEK.K** |
| **27** | **73983760** | **Stress-induced-phosphoprotein 1 (STI1)** | **STIP1** | **63** | **6.35** | **73** | **7** | **13** | **1131.6400  1487.7871  1034.4702  1072.4252  1888.8481  1001.5579  1099.6423** | **R.KAAALEFLNR.F  R.LAYINPDLALEEK.N  K.ALDLDSNCK.E  R.CMMAQYNR.H  R.AMADPEVQQIMSDPAMR.L  R.LILEQMQK.D  K.LMDVGLIAIR.-** |
| **28** | **114593211** | **WD repeat-containing protein 1 isoform 8** | **WDR1** | **66** | **6.68** | **137** | **11** | **29** | **1272.7190  1144.6240  2417.2339  1617.7674  1101.5131   2042.9043   1206.6145   1718.8151   2584.2193   2279.0528   2539.2131** | **K.KVFASLPQVER.G  K.VFASLPQVER.G  R.NIDNPALADIYTEHAHQVVVAK.Y  K.YAPSGFYIASGDVSGK.L  K.YEYQPFAGK.I  R.LATGSDDNCAAFFEGPPFK.F  K.FKFTIGDHSR.F  R.FATASADGQIYIYDGK.T  K.AHDGGIYAISWSPDSTHLLSASGDK.T K.GPVTDVAYSHDGAFLAVCDASK.V  K.VVTVFSVADGYSENNVFYGHHAK.I** |
| **29** | **115496400** | **dihydropyrimidinase-like 2** | **DPYSL2** | **63** | **5.95** | **157** | **12** | **34** | **2376.1669 2899.5038 1835.9451 1014.5458 2147.0494 1724.8039 2085.8884 1083.6288 1681.8634 1139.6015 1619.7865 2168.0610** | **R.DIGAIAQVHAENGDIIAEEQQR.I  R.ILDLGITGPEGHVLSRPEEVEAEAVNR.S  R.SITIANQTNCPLYITK.V  K.SAAEVIAQAR.K  K.AVGKDNFTLIPEGTNGTEER.M  K.MDENQFVAVTSTNAAK.V  K.THNSSLEYNIFEGMECR.G  R.GSPLVVISQGK.I  K.IVLEDGTLHVTEGSGR.Y  R.KPFPDFVYK.R  R.GLYDGPVCEVSVTPK.T  R.NLHQSGFSLSGAQIDDNIPR.R** |
| **32** | **119597640** | **protein disulfide isomerase family A, member 3** | **PDIA3** | **54** | **6.78** | **99** | **8** | **20** | **1171.5331 1340.6764 1369.6877 1514.7517 1618.7766 1644.8736 2574.2965 2702.3914** | **K.FVMQEEFSR.D  R.GFPTIYFSPANK.K  R.ELSDFISYLQR.E  R.FLQDYFDGNLKR.Y  K.DLLIAYYDVDYEK.N  K.FLDAGHKLNFAVASR.K  K.TFSHELSDFGLESTAGEIPVVAIR.T  R.KTFSHELSDFGLESTAGEIPVVAIR.T** |
| **33** | **54035918** | **Coronin-1B (Coroninse)** | **CORO1B** | **54** | **5.85** | **133** | **11** | **22** | **910.5024   913.4657   1028.5614   1039.6026   1169.6516  1213.6720   1235.6775   1328.6944  1523.7078   1940.9744  2103.9796** | **R.HVFGQPVK.N  K.VFTTGFSR.M  R.ALVKEQGER.I  R.DAGPVLISLR.E  R.IGRLEEQLGR.V  K.FRHVFGQPVK.N  R.VGIVTWHPTAR.N  K.CEPIVMTVPRK.S  R.VTWDSTFCAVNPK.F  R.LDSLHPDLIYNVSWNR.N  R.HVFGQPVKNDQCYEDIR.V** |
| **37** | **114657259** | **aldehyde dehydrogenase 1A2 isoform 1** | **ALDH1A2** | **45** | **5.57** | **153** | **10** | **27** | **937.4327  1134.6185  1266.6278  1426.8031  1658.8191  1814.9202  1973.9192  2008.9853  2056.0476  2102.0142** | **R.EMGEFGLR.E  R.LAFSLGSVWR.R  R.FKTMDEVIER.A  K.ILELIQSGVAEGAK.L  R.IFVEESIYEEFVR.R  R.IFVEESIYEEFVRR.S  K.GFFIEPTVFSNVTDDMR.I  R.ANNSDFGLVAAVFTNDINK.A R.IVGSPFDPTTEQGPQIDKK.Q  R.KGFFIEPTVFSNVTDDMR.I** |
| **38** | **860908** | **Vimentin** | **VIM** | **45** | **4.75** | **136** | **12** | **38** | **1092.5199  1120.5764  1124.5978  1294.6591  1308.5986  1569.8878  1681.8522  1687.8199  1733.8076  1837.9534  2323.1114  2376.1590** | **K.FADLSEAANR.N  R.EYQDLLNVK.M  R.FANYIDKVR.F  K.MALDIEIATYR.K  K.NLQEAEEWYK.S  R.ISLPLPNFSSLNLR.E  R.ETNLESLPLVDTHSK.R  R.VEVERDNLAEDIMR.L  R.LQDEIQNMKEEMAR.H  R.ETNLESLPLVDTHSKR.T  K.LQEEMLQREEAESTLQSFR.Q  R.QVQSLTCEVDALKGTNESLER.Q** |
| **39** | **119617634** | **chaperonin containing TCP1, subunit 2 (beta)** | **CCT2** | **46** | **6.09** | **118** | **9** | **30** | **1958.9697  1375.7361  2040.0415  2040.0415  1531.8279  1129.5451  2096.1153  2346.1889  1581.9089** | **R.EALLSSAVDHGSDEVKFR.Q  K.LLTHHKDHFTK.L  K.LGGSLADSYLDEGFLLDKK.I  K.LGGSLADSYLDEGFLLDKK.I  K.ILIANTGMDTDKIK.I  K.HGINCFINR.Q  R.LALVTGGEIASTFDHPELVK.L  R.MLPTIIADNAGYDSADLVAQLR.A  R.QVLLSAAEAAEVILR.V** |
| **40** | **51863477** | **glutamate dehydrogenase** | **GLUD1** | **56** | **6.71** | **231** | **16** | **35** | **955.5127   962.5185   999.4483  1058.5257  1195.6713  1218.6066  1424.6208  1490.7187  1542.8253  1736.8845  1914.9145  1919.9047  1957.0308  2043.0094  2163.0629  2241.1640** | **R.LTFKYER.D  K.YNLGLDLR.T  K.MVEGFFDR.G  K.NLNHVSYGR.L  K.LQHGSILGFPK.A  K.CAVVDVPFGGAK.A  R.DDGSWEVIEGYR.A  K.DIVHSGLAYTMER.S  R.GASIVEDKLVEDLR.T  K.HGGTIPIVPTAEFQDR.I  K.GFIGPGIDVPAPDMSTGER.E  R.DSNYHLLMSVQESLER.K  K.ELEDFKLQHGSILGFPK.A  K.KGFIGPGIDVPAPDMSTGER.E  R.ISGASEKDIVHSGLAYTMER.S  K.IIAEGANGPTTPEADKIFLER.N** |
| **41** | **42558920** | **Aldehyde dehydrogenase family 1 member A1** | **ALDH1A1** | **55** | **6.99** | **188** | **13** | **32** | **944.5291  1099.5985  1114.6597  1188.6040  1359.7431  1423.6666  1556.7834  1639.6469  1644.8035  1655.8042  1800.9046  2002.9458  2713.3710** | **K.SLDDVIKR.A  K.LADLIERDR.L  K.ILDLIESGKK.E  R.QAFQIGSPWR.T  R.LLLATMESLNAGK.L  R.YCAGWADKIQGR.T  R.ANNTTYGLSAGIFTK.D  R.TMPMDGDFFCYTR.H  R.LFVEESIYDEFVR.R  R.ELGEYGLQQYTEVK.T  R.LFVEESIYDEFVRR.S  K.GYFIQPTVFSNVTDEMR.I  K.YVLGNPLAPEVNQGPQIDKEQYNK.I** |
| **42** | **27448554** | **septin SEPT8_v3** | **08-Sep** | **53** | **6.22** | **77** | **7** | **20** | **979.6430  1020.5240  1152.5200  1308.6826  1931.0112  2055.0636  2347.0691** | **K.VNIIPIIAK.A  K.EFLSELQR.K  R.SLFDYHDTR.I  R.ELHEKFEHLK.R  R.LRPQTYDLQESNVQLK.L  R.SLSLGGHVGFDSLPDQLVSK.S  R.QYPWGVVQVENENHCDFVK.L** |
| **44** | **73995214** | **Aldehyde dehydrogenase, mitochondrial precursor** | **ALDH2** | **57** | **6.63** | **114** | **9** | **18** | **1039.5702  1131.5825  1287.6836  1402.7530  1457.6727  1598.7827  1730.8151  2449.1397  2577.2346** | **K.YGLAAAVFTK.D  R.AAFQLGSPWR.R  R.AAFQLGSPWRR.M  K.EEIFGPVMQILK.F  R.YYAGWADKYHGK.T  R.ELGEYGLQAYTEVK.T  R.TFVQEDVYAEFVER.S  R.VVGNPFDSQTEQGPQVDETQFK.K  R.VVGNPFDSQTEQGPQVDETQFKK.I** |
| **48** | **49870** | **alpha-actin (AA 27-375) [Mus musculus]** | **ACTC1** | **39** | **5.83** | **88** | **5** | **18** | **922.5600  1129.5403  1514.7419  1789.8846  2227.0579** | **K.IIAPPERK.Y  R.GYSFVTTAER.E  K.IWHHTFYNELR.V  K.SYELPDGQVITIGNER.F  K.DLYANNVLSGGTTMYPGIADR.M** |
| **50** | **232037** | **Elongation factor 1-gamma (EF-1-gamma)** | **EEF1G** | **50** | **6.31** | **101** | **7** | **19** | **1346.7306  1091.6702   974.5185  1122.6185  1240.6451  2686.2339  1667.7732** | **K.ALIAAQYSGAQVR.V  R.ILGLLDAHLK.T  K.QVLEPSFR.Q  K.AKDPFAHLPK.S  K.STFVLDEFKR.K  R.GQELAFPLSPDWQVDYESYTWR.K  R.EYFAWEGAFQHVGK.A** |
| **54** | **178027** | **actin gamma 2** | **ACTG2** | **42** | **5.23** | **89** | **7** | **21** | **922.5600  1035.6440  1129.5403  1499.7004  1500.7262  1789.8846  2227.0579** | **K.IIAPPERK.Y  K.IKIIAPPER.K  R.GYSFVTTAER.E  K.QEYDEAGPSIVHR.K  K.IWHHSFYNELR.V  K.SYELPDGQVITIGNER.F  K.DLYANNVLSGGTTMYPGIADR.M** |
| **56** | **381964** | **actin-related protein 1, centractin alpha** | **ACTR1A** | **43** | **6.59** | **98** | **7** | **28** | **1399.6706  1516.7959  2018.8978  1085.5546  2533.2812  1772.7893  2138.0201** | **K.YCFPNYVGRPK.H  R.VMAGALEGDIFIGPK.A  R.YPMEHGIVKDWNDMER.I  R.IWQYVYSK.D  K.DQLQTFSEEHPVLLTEAPLNPR.K  K.EGYDFHSSSEFEIVK.A  R.ACYLSINPQKDETLETEK.A** |
| **57** | **29126784** | **Actin-related protein 2 homolog** | **ACTR2** | **45** | **6.31** | **88** | **7** | **24** | **1211.5393   1391.6510   1612.7270   1642.7661   1750.8382   1770.9086   2087.0568** | **K.DKDNFWMTR.Q  K.HLWDYTFGPEK.L  R.GYAFNHSADFETVR.M  K.LCYVGYNIEQEQK.L  R.SMLEVNYPMENGIVR.N  K.HIVLSGGSTMYPGLPSR.L  K.VGNIEIKDLMVGDEASELR.S** |
| **58** | **25282395** | **glutathione-S-transferase, mu 5** | **GSTM5** | **27** | **5.38** | **118** | **8** | **39** | **938.5225  1014.5822  1134.5379  1509.6955  1765.8596  1778.8807  1815.8106  1957.9785** | **K.QFSLFLGK.F  K.ITQSNAILR.Y  K.CLDEFPNLK.A  R.VDIMENQIMDFR.I  K.LDLDFPNLPYLMDGK.N Oxidation (M)  R.IRVDIMENQIMDFR.I  K.CLDEFPNLKAFMCR.F Oxidation (M)  K.LTFVDFLTYDVLDQNR.M** |
| **59** | **1703316** | **Annexin A1 (Annexin I)** | **ANXA1** | **39** | **6.28** | **99** | **8** | **27** | **943.5352  1261.5939  1370.7656  1386.7606  1542.8617  1701.8784  2111.9799  2188.0357** | **R.SYLHLRR.V  K.TPAQFDADELR.A  K.VLDLELKGDIEK.C  K.GVDEATIIDILTK.R  K.GVDEATIIDILTKR.N  K.GLGTDEDTLIEILASR.N  K.QAWFIDNEEQDYINTVK.T  K.YGVSLCQAILDETKGDYEK.I** |
| **60** | **109102505** | **PP1-beta catalytic subunit (PP-1B) isoform 1** | **PPP1CB** | **35** | **5.82** | **125** | **8** | **31** | **1040.5073  1174.6016  1193.5063  1438.7972  1570.8038  1659.8039  1760.8958  2581.2164** | **R.HDLDLICR.A  K.IVQMTEAEVR.G  R.IYGFYDECK.R  K.IKYPENFFLLR.G  K.FLNRHDLDLICR.A  K.ICGDIHGQYTDLLR.L  K.YQYGGLNSGRPVTPPR.T  R.LFEYGGFPPEANYLFLGDYVDR.G** |
| **61** | **30794280** | **Albumin** | **ALB** | **71** | **5.82** | **78** | **8** | **15** | **926.4861  1438.8044  1478.7881  1566.7354  1594.9194  1638.9304  1879.9138  2044.0206** | **K.YLYEIAR.R  R.RHPEYAVSVLLR.L  K.LGEYGFQNALIVR.Y  K.DAFLGSFLYEYSR.R  R.HPEYAVSVLLRLAK.E  R.KVPQVSTPTLVEVSR.S  R.RPCFSALTPDETYVPK.A  R.RHPYFYAPELLYYANK.Y** |
| **62** | **119615295** | **capping protein (actin filament) muscle Z-line** | **CAPZB** | **30** | **5.88** | **146** | **10** | **47** | **1107.6400   1170.5920 1230.5339  1245.6387  1336.6405  1684.7879 1695.8216 1923.8057 2217.0160  2286.0368** | **R.RLPPQQIEK.N  R.STLNEIYFGK.T  K.DYLLCDYNR.D  R.LVEDMENKIR.S  K.SGSGTMNLGGSLTR.Q  K.GCWDSIHVVEVQEK.S  R.KLEVEANNAFDQYR.D  K.DYLLCDYNRDGDSYR.S  R.SPWSNKYDPPLEDGAMPSAR.L  R.QMEKDETVSDCSPHIANIGR.L** |
| **78** | **157821497** | **NADH dehydrogenase (ubiquinone) Fe-S protein 8** | **NDUFS8** | **24** | **5.53** | **72** | **8** | **40** | **1207.6423  1223.6373  1256.6587  1272.6536  1914.9839   984.5253  2040.9972  2679.3292** | **R.ILMWTELFR.G  R.ILMWTELFR.G Oxidation (M)  R.GLGMTLSYLFR.E  R.GLGMTLSYLFR.E Oxidation (M)  R.EPATINYPFEKGPLSPR.F  R.FRGEHALR.R  K.LCEAICPAQAITIEAEPR.A  K.LLNNGDKWEAEIAANIQADYLYR.-** |

**GI^1^: Genebank ID, Mw^2^: relative molecular weight, Pi^3^: calculated isoelectric point, Score^4^: Mowse score, Pep^5^: Queries matched peptide number, Cov^6^: coverage sequence percent (%), * : identified by MS/MS, Pep. Mr^7^: Calculated relative molecular mass of the matched peptide.**
